# Ketogenic Nutrition in Combination With PPARα Activation Induced Metabolic Failure and Exacerbated Muscle Weakness in Septic Mice

**DOI:** 10.1002/jcsm.70156

**Published:** 2025-12-09

**Authors:** Caroline Lauwers, Wouter Vankrunkelsven, Sarah Derde, Sarah Vander Perre, Inge Derese, Lies Pauwels, Ilse Vanhorebeek, Louis Libbrecht, Pieter Vermeersch, Jan Gunst, Greet Van den Berghe, Michael P. Casaer, Lies Langouche

**Affiliations:** ^1^ Department of Cellular and Molecular Medicine, Laboratory and Clinical Division of Intensive Care Medicine KU Leuven Leuven Belgium; ^2^ Laboratory of Hepatology KU Leuven Leuven Belgium; ^3^ Clinical Department of Laboratory Medicine UZ Leuven Leuven Belgium

**Keywords:** critical illness, ketone bodies, long‐chain triglycerides, metabolism, muscle weakness, PPARα, sepsis

## Abstract

**Background:**

Suppression of the peroxisome proliferator‐activated receptor alpha (PPARα) has been related to poor outcomes in sepsis and may compromise ketogenesis during critical illness. Infusion of 3‐hydroxybutyrate (3HB) was shown to attenuate muscle weakness in septic mice. We hypothesise that endogenous ketogenesis induced by pharmacological PPARα activation, either alone or combined with ketogenic nutrition, is safe and can also mitigate muscle weakness in septic mice.

**Methods:**

In a fluid‐resuscitated, antibiotic‐treated mouse model of prolonged sepsis, we first (Study 1) assessed the safety and effectiveness (impact on ketosis and muscle weakness) of the PPARα agonist pemafibrate (1 mg/kg/d, *n* = 16), versus placebo (*n* = 15) combined with standard balanced parenteral nutrition (PN), composed of glucose, amino acids and long‐chain triglycerides (LCT) (balanced‐TPN). We subsequently (Study 2) evaluated the impact of pemafibrate combined with four types of PN on ketosis and muscle weakness: balanced‐TPN (*n* = 18), TPN with extra LCTs (TPN + LCT, n = 18), low‐dose pure LCT emulsion (Low‐LCT, *n* = 16) and high‐dose pure LCT emulsion (High‐LCT *n* = 18). Carbohydrates and amino acids were omitted in the pure LCT groups. Healthy control mice (HC, *n* = 19) served as controls. Ex vivo muscle force was measured as the primary outcome. Metabolic, inflammatory and microstructural parameters were assessed on plasma and in muscle and liver tissue by targeted metabolomics, gene expression analysis, biochemical and metabolite assays and histological assessment.

**Results:**

Pemafibrate treatment with balanced‐TPN upregulated hepatic gene expression of PPARα (*Ppara*) and its downstream genes (*Cd36*, *Cpt1a*, *Atgl*, *Acadl, Hadha, Acox1,* and *Hmgcs2*) (*p* < 0.0001) and was well tolerated. However, pemafibrate treatment with the use of balanced‐TPN administration did not induce detectable ketosis or improve muscle weakness. In combination with pemafibrate, TPN + LCT also did not induce ketosis, nor did it affect muscle weakness. In contrast, 3‐hydroxybutyrate plasma concentrations increased with High‐LCT (95‐fold) and Low‐LCT (10‐fold) (*p* < 0.0001) in combination with pemafibrate, yet muscle force declined further (High‐LCT 25.0%, Low‐LCT 10.7% of HC, *p* < 0.0001). Blood glucose was lowered with pure High‐LCT and Low‐LCT (High‐LCT 86.9%, Low‐LCT 55.1% of TPN, *p* < 0.05), while plasma lipids and LC‐carnitines were increased (*p* < 0.0001). Markers of hepatic protein catabolism were upregulated with High‐LCT and Low‐LCT (*p* < 0.007), while muscle glycolytic intermediates (*p* < 0.0001) and ATP levels (*p* < 0.0001) were depleted.

**Conclusions:**

In septic mice, pemafibrate combined with balanced‐TPN or lipid‐rich TPN induced PPARα activation but did not result in ketosis nor affect muscle weakness. Pemafibrate combined with pure LCTs induced ketosis in sepsis but worsened muscle weakness, possibly explained by muscular bioenergetic failure.

**Abbreviations**
3HB: 3‐beta‐hydroxybutyrate
AA: amino acids
Aass: aminoadipate‐semialdehyde synthase
AcAc: acetoacetate
Acadl: acyl‐coenzyme A dehydrogenase, long‐chain
Acox1: acyl‐coenzyme A oxidase 1, palmitoyl
Atgl: adipose triglyceride lipase
ATP: adenosine triphosphate
Bdh1: 3‐hydroxybutyrate dehydrogenase, type 1
Cd36: CD36 molecule
CLP: caecal ligation and puncture
CoA: coenzyme A
Cpt1a: carnitine palmitoyl transferase 1a
Cpt1b: carnitine palmitoyl transferase 1b
Cs: citrate synthase
DHAP: dihydroxyacetone phosphate
DMSO: dimethyl sulfoxide
EDL: m. extensor digitorum longus
Fabp3: fatty acid binding protein 3, muscle and heart
Fbp1: fructose bisphosphatase 1
Fbxo32: F‐Box Protein 32
FFA: free fatty acids
GADP: glyceraldehyde‐3‐phosphate
GGT: γ‐glutamyltransferase
Got1: glutamic‐oxaloacetic transaminase 1
Hadha: hydroxyacyl‐CoA dehydrogenase trifunctional multienzyme complex subunit alpha
HC: healthy control
Hk1: hexokinase 1
Hmgcs2: 3‐hydroxy‐3‐methylglutaryl‐CoA synthase 2
H&E: haematoxylin and eosin
ICUAW: intensive care unit‐acquired weakness
Idh2: isocitrate dehydrogenase 2 (NADP+), mitochondrial
Il6: interleukin 6
LC–MS–MS: liquid chromatography with tandem mass spectrometry
Ldha: lactate dehydrogenase a
LCT: long‐chain triglycerides
MDA: malondialdehyde
mRNA: messenger RNA
Mstn: myostatin
Myf5: myogenic Factor 5
Myod1: myogenic differentiation 1
Myog: myogenin
Oat: ornithine aminotransferase
Ogdh: oxoglutarate (alpha‐ketoglutarate) dehydrogenase (lipoamide)
Oxct1: 3‐oxoacid CoA transferase 1
OXPHOS: oxidative phosphorylation
Pax7: paired box 7
Pck1: phosphoenolpyruvate carboxykinase 1, cytosolic
Pcx: pyruvate carboxylase
Pdha1: pyruvate dehydrogenase E1 alpha 1
Pdk4: pyruvate dehydrogenase kinase, isoenzyme 4
PEG: polyethylene glycol
PEP: phosphoenolpyruvate
PF: pemafibrate
Pfkfb3: 6‐phosphofructo‐2‐kinase/fructose‐2,6‐biphosphatase 3
Pfkm: phosphofructokinase, muscle
Pkm: pyruvate kinase, muscle
Ppara: peroxisome proliferator activated receptor alpha
PPARα: peroxisome proliferator‐activated receptor alpha
Rn18s: 18S ribosomal RNA
SCOT: succinyl‐CoA 3‐oxoacid CoA‐transferase 1
Sdha: succinate dehydrogenase complex, subunit A, flavoprotein (Fp)
Sds: serine Dehydratase
TCA: tricarboxylic acid cycle
TG: triglycerides
Tnf: tumour necrosis factor
TPN: total parenteral nutrition
Trim63: tripartite motif containing 63
Ubb: ubiquitin B
Ubc: ubiquitin C
UCP: uncoupling protein

## 1 Introduction

Intensive care unit‐acquired weakness (ICUAW) affects over 40% of critically ill patients and has been associated with the risk of death, delayed recovery and poor long‐term quality of life [1]. The exact pathophysiology remains incompletely understood but involves inflammation, metabolic alterations, cellular dysfunction and immobilisation [1, 2]. Currently, therapeutic options are lacking, emphasising the need for better preventive strategies [3, 4]. One such preventive strategy is accepting a macronutrient deficit during the first week in ICU, which has been shown to reduce ICUAW and accelerate recovery [3]. This benefit could partly be attributed to the fasting response that shifts metabolism towards lipid oxidation and ketogenesis, enhances cellular repair mechanisms and induces stress resistance [3, 4, 5]. However, during critical illness, fasting‐induced ketogenesis appears to be impaired [6, 7], possibly related to suppressed expression of the nuclear receptor peroxisome proliferator‐activated receptor α (PPARα), a key regulator of lipid and ketone body metabolism [8, 9]. Indeed, hepatic PPARα expression was found to be suppressed during sepsis, and, in critically ill children, suppression of whole blood PPARα was in proportion to the severity of illness [10, 11]. Moreover, in animal models of sepsis, knockout of PPARα dysregulated the immune response and increased mortality, whereas PPARα agonists improved organ function and decreased mortality [10, 12, 13]. These studies, however, did not investigate the impact on ketogenesis and muscle weakness.

Pharmacological PPARα agonists such as fibrates may improve the lipid profile of human subjects by activating lipid transport and oxidative pathways and by stimulating ketogenesis [14, 15]. Currently, it remains uncertain whether pharmacological activation of PPARα may promote ketogenesis and affect muscle weakness during sepsis. Alternatively, ketosis can also be obtained with ketogenic diets that are low in carbohydrates and high in lipids. These ketogenic diets are sometimes used in the ICU for refractory epilepsy and may attenuate hyperglycaemia [16, 17] and facilitate weaning from mechanical ventilation in specific patient populations [18, 19]. The metabolic impact of ketogenic diets in critically ill patients remains poorly understood, however, especially in the absence of amino acids and carbohydrates, and current international nutritional guidelines do not recommend such nutritional strategies [20, 21]. The current study, performed in a standardised murine model of sepsis‐induced critical illness, aimed to investigate whether pharmacological activation of PPARα, either alone or in combination with ketogenic nutritional formulae, can overcome impaired ketogenesis, enhance lipid metabolism and prevent muscle weakness. A more in‐depth metabolic workup of liver and muscle tissue was included to understand the pathophysiologic basis of the findings.

## 2 Methods

### 2.1 Animal Study Design

A standardised centrally catheterised murine sepsis model, previously described in detail, was used for two experiments [22]. In brief, a central venous catheter was placed in the central jugular vein and polymicrobial abdominal sepsis was induced by a 50% caecal ligation and puncture (CLP) with an 18G needle in 24‐week‐old C57Bl/6JRj male mice (Janvier, Le Genest‐Saint‐Isle, France). During the first 20 h, a fluid resuscitation (Plasmalyte 50%, Baxter, Volulyte 50%, 10 mL/kg/h, Fresenius Kabi) was provided without intravenous macronutrients to comply with the current practice of withholding parenteral nutrition (PN) in the acute phase of illness. From Day 1 of sepsis onwards, mice received PN in a dose covering 40% of the caloric intake of healthy mice until the end of the experiment. PN was administered continuously at a rate of 0.2 mL/h. Pain/discomfort was assessed twice daily by the Mouse Grimace Pain Score [23], and healthy control mice fed ad libitum served as a reference. Both experiments ended 5 days after CLP, and ex vivo muscle force was measured as the primary outcome. Analyses on plasma and tissue samples were conducted in all mice surviving until the end of the experiment, unless stated otherwise in Supplementary Table S1 for Study 1 and Supplementary Table S2 for Study 2. Additional background on the experimental setup can be found in the supplemental information. Both studies were approved by the local Ethical Committee (P180‐2016; P097/2021).

#### 2.1.1 Study 1: Safety and Ketogenic Efficacy of Pharmacological PPARα Activation by Pemafibrate (PF) With State of the Art Balanced PN in Murine Sepsis

To assess the safety and effectiveness of pharmacological PPARα activation, the selective PPARα agonist PF was first tested in septic mice receiving the standard of care PN (balanced‐TPN, composed of glucose, amino acids and long‐chain triglycerides (LCT), Olimel N7E, Baxter) (Supplementary Figure S1). Starting 1 h before CLP, TPN‐treated septic mice were randomised to receive either PF (total dose of 1 mg/kg/day, dissolved in 0.5% DMSO (dimethylsulfoxide), 2% PEG (polyethyleneglycol) 300, 0.25% Tween80, 97.25% plasmalyte, 400 μL per injection, Biorbyt) or the vehicle as placebo subcutaneously twice daily during the study (Supplementary Table S3).

#### 2.1.2 Study 2: Ketogenic Efficacy and Impact on Muscle Weakness of Pharmacological PPARα Activation by PF in Combination With Various IV Ketogenic Formulae in Murine Sepsis

In the second study, all septic mice received the selective PPARα agonist PF (as described in study 1) (Supplementary Figure S1). After the initial fluid resuscitation, septic mice were randomised to receive various types of PN: (1) the standard balanced‐TPN (Olimel N7E, Baxter, Lessines, Belgium; 51.0% of kcal glucose, 32.8% of kcal LCT and 16.2% of kcal AA, 5.3 kcal/d); (2) balanced‐TPN with an extra LCT emulsion (TPN + LCT), isocaloric to the amount of LCT already present in the balanced‐TPN (1.7 kcal/day) to increase available lipids; (3( a pure LCT emulsion (Clinoleic 20%, Baxter, Lessines, Belgium) at a low dose (Low‐LCT), isocaloric to the lipid content of the standard balanced‐TPN (1.7 kcal/day) or (4) a pure LCT emulsion at a high dose (High‐LCT), isocaloric to the balanced‐TPN (5.3 kcal/day) (Supplementary Figure S1, Supplementary Table S3). These pure Low‐LCT and High‐LCT groups received no amino acids or carbohydrates to prevent their potentially inhibiting effects on ketogenesis. All PN solutions were administered in an isovolumetric manner among the groups, and electrolytes were adjusted accordingly (Supplementary Table S3).

### 2.2 Ex Vivo Muscle Force Measurements

In skeletal muscle harvested from mice who survived until the end of the experiment, muscle force was measured ex vivo as the primary endpoint. The hindlimb m. extensor digitorum longus (EDL) was carefully dissected, directly after sacrifice, and suspended in a temperature‐controlled (30°C) organ bath filled with HEPES‐fortified Krebs‐Ringer solution (300C‐LR Dual‐Mode muscle lever, Aurora Scientific, Ontario, Canada). Starting from the resting length, maximal isometric tetanic force was measured as the average force generated by three consecutive tetanic stimuli (180 Hz stimulation frequency, 200 ms duration, 0.2 ms pulse width, 2 min rest intervals). Additionally, the specific maximal isometric tetanic force was calculated, dividing the maximal isometric tetanic force by the muscle cross‐sectional area. The fatigue index, a measure of muscle endurance, was calculated as the ratio of the force generated by the third tetanic stimulus divided by the force of the first stimulus.

### 2.3 Whole Blood and Plasma Analyses

Blood glucose and 3‐hydroxybutyrate (3HB) concentrations were measured by point‐of‐care testers (Accu‐Check (Roche, Basel, Switzerland) and StatStrip Xpress 2 (nova biomedical, Waltham, MA) respectively) on whole blood drawn from the tail vein at the start of the experiment, on the morning of Day 1 (before the initiation of PN) and on the evening of Day 3. Commercial assays were used to measure plasma concentrations of free fatty acids (FFAs, Cayman Chem, Ann Arbor, MI), triglycerides (TG, Abcam, Cambridge, United Kingdom), LDL and HDL cholesterol (Diazyme, Poway, CA, United States), γ‐glutamyltransferase (GGT) (Sigma‐Aldrich, St. Louis, United States), interleukin 6 (R&D Systems, Abingdon, United Kingdom), tumour necrosis factor α (R&D Systems, Abingdon, United Kingdom), urea (Invitrogen, Waltham, United States) and malondialdehyde (MDA, Abcam, Cambridge, United Kingdom). Plasma 3HB after 5 days of sepsis was quantified with an internally developed enzymatic assay [24]. Plasma acylcarnitine profile and free carnitine levels were assessed by liquid chromatography with tandem mass spectrometry (LC–MS–MS) in collaboration with the clinical department of laboratory medicine, UZ Leuven.

### 2.4 Tissue Analyses

The m. tibialis anterior was used for histology and mitochondrial measurements, and the larger gastrocnemius muscle was homogenised and used for local gene expression and metabolite analyses. The water content of liver and muscle tissue was analysed by a freeze‐drying process (frozen tissue samples were dried for 3 h at 100°C). RNA was isolated from liver and muscle tissue with Qiazol and the RNeasy mini RNA isolation kit (QIAGEN, Venlo, The Netherlands). DNAse treatment was applied to remove genomic DNA, and RNA was reverse transcribed with the use of random hexamers. Relative gene expression was determined with the 2−ΔΔCt method with 18S ribosomal RNA (*Rn18s*) as the housekeeping gene for liver tissue and succinate dehydrogenase complex flavoprotein subunit A (*SDHA*) for muscle tissue by commercial TaqMan assays (Applied Biosystems, Carlsbad, CA, United States) (Supplementary Figure S2; Supplementary Table S4). Muscle and liver triglycerides were measured with Triglycerides Reagent (Thermo Fisher, Middletown, United States) after hexane extraction. Commercial assays (Abcam, Cambridge, United Kingdom) were used to measure cholesterol and glycogen content in liver and muscle homogenates. Citrate synthase and mitochondrial respiratory chain complex I and V activities in m. tibialis anterior muscle were assessed by spectrophotometry at 30°C as described previously [25]. Immunoblotting was conducted for uncoupling protein 3 (UCP3) on gastrocnemius muscle in the second study (#PA1‐055, Invitrogen, Waltham, MA, United States). Tissue samples were homogenised in sample buffer (1% Nonidet P‐40; 10% glycerol; 20 mM Tris–HCl (pH = 7.6); 10 mM EDTA; 1x Complete Mini). The protein content was determined with Coomassie Protein Assay Reagent (Pierce Biotechnology Inc.) using a standard curve of BSA. Western blots were performed using commercial 4%–20% tris‐glycine gels (Biorad, Hercules, CA) and PVDF membranes (Thermo Fisher Scientific, Waltham, MA, United States). Secondary horseradish peroxidase–conjugated antibodies were purchased from DakoCytomation (Heverlee, Belgium). Blots were developed with the Western Lightning chemiluminescence reagent Plus kit (Perkin Elmer, Zaventem, Belgium), visualised with the G:BOX Chemi XRQ (SynGene, Cambridge, United Kingdom) and analysed with the SynGene software.

### 2.5 Histology

Cross‐sectional paraffin liver sections were stained by haematoxylin and eosin (H&E) and scored semiquantitatively for inflammation, hypertrophy, necrosis and steatosis. A steatosis score [4] was calculated and included the severity of inflammation, microvesicular and macrovesicular fatty change and the presence of ballooning hepatocytes. H&E stained muscle paraffin sections were assessed for fibre size, fibre shape, the presence of fibrous tissue, inflammation, necrosis and internalised nuclei.

### 2.6 Metabolomics

Targeted metabolomics of muscle and liver tissue was performed with a Dionex UltiMate 3000 LC System (Thermo Scientific Bremen, Germany) equipped with a C‐18 column (Acquity UPLC ‐HSS T3 1. 8 μm; 2.1 × 150 mm, Waters) coupled to a Q Exactive Orbitrap mass spectrometer (Thermo Scientific) operating in negative ion mode in collaboration with the metabolomics expertise centre (KU Leuven). The Xcalibur software (Thermo Scientific) was used for data collection. For data analysis, peak areas were integrated (El‐Maven—Polly—Elucidata). Data analysis of metabolomics data was done by the MetaboAnalystR package [26]. Pathway analysis was conducted (more information can be found in the supporting information), and correlations were calculated between metabolites by Spearman correlation. Only significant (*p* < 0.05) correlations were reported.

### 2.7 Statistical Analyses

Data were summarised as mean and standard deviation, or median and interquartile range, depending on their distribution. Normality was assessed visually and by the Shapiro–Wilk test. When data were distributed normally, data were compared by analysis of variance (ANOVA) in the case of multiple groups or Student's *t*‐test in the case of two groups, or the Kruskal–Wallis test or Wilcoxon signed‐rank test, respectively, when data were not distributed normally. Kaplan–Meier survival curves were assessed by the log‐rank test. Two‐sided *p*‐values ≤ 0.05 were considered statistically significant. Pathway analysis was corrected for multiple testing by the false discovery rate (FDR). Other univariate analyses were not corrected for multiple testing. Continuous data are represented by boxplots of which the whiskers extend until the furthest point within 1.5 times the interquartile range. Statistical analyses were performed with the R statistical programming language (R Core Team (2023). _R: A Language and Environment for Statistical Computing_. R Foundation for Statistical Computing, Vienna, Austria <https://www.R‐project.org/> (version 4.3.2)) and the RStudio interface (version 2024.04.0, Boston, MA, United States).

## 3 Results

### 3.1 Assessment of Safety and Ketogenic Effectiveness of the Pharmacological PPARα Agonist, PF, With State of the Art Balanced PN in Murine Sepsis (Study 1)

**FIGURE 1:**
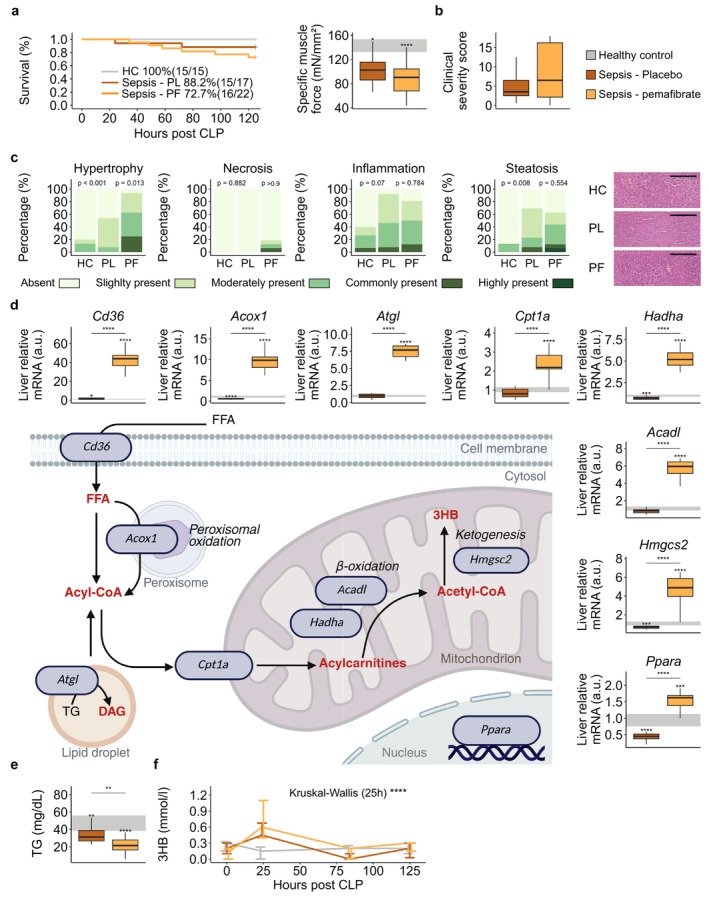
Impact of pharmacological PPARα activation on the metabolic alterations during sepsis. (a) Survival after 5 days of sepsis and specific maximal muscle force. (b) Cumulative pain score of surviving septic mice. (c) Semiquantitative scoring of liver histology including signs of hypertrophy, necrosis, inflammation and steatosis. Representative H&E images are shown per group. (d) Relative mRNA expression of PPARα and its downstream genes in the liver. (e) Plasma concentrations of triglycerides. (f) Blood concentrations of 3‐hydroxybutyrate. Healthy mice *n* = 15; septic placebo‐treated mice *n* = 15; septic pemafibrate‐treated mice *n* = 16. Abbreviations: a.u., arbitrary unit; DAG, diacylglycerol; HC, healthy control; PF, pemafibrate; PL, placebo; rel., relative; TAG, triacylglycerol. The interquartile range of HC mice is shown in grey, and asterisks above boxplots denote comparisons with HC mice. Statistical significance is shown by */**/***/****: *p* < 0.05/0.01/0.001/0.0001. Black scale bars in histological images denote 200 μm. In the line plot, the overall statistical difference was assessed by the Kruskal–Wallis test for each time point (0, 25, 84 and 125 h). Illustrations were created with BioRender.com. For histological analyses, left‐sided *p*‐values denoted overall statistical comparison assessed by the Kruskal–Wallis test among all groups and right‐sided *p*‐values among the septic mice.

PF treatment did not affect survival rates among septic mice (*p* = 0.3) or muscle force and clinical severity scores throughout the 5 days of sepsis during balanced‐TPN infusion as compared with placebo‐treated mice (Figure 1a,b). PF induced liver hypertrophy [27] but did not further increase signs of hepatic necrosis, inflammation or steatosis compared with placebo (Figure 1c). Compared with placebo, PF treatment strongly increased hepatic mRNA expression of *Ppara* and its downstream genes involved in fatty acid oxidation and ketogenesis (*Cd36*, *Acox1*, *Atgl*, *Cpt1a*, *Hadha*, *Acadl* and *Hmgcs2*) (Figure 1d) and further lowered plasma TG concentrations (Figure 1e). Yet, PF administration was unable to affect 3HB blood levels during 5 days of sepsis (Figure 1f) in comparison with placebo‐treated septic mice.

### 3.2 Assessment of Ketogenic Effectiveness and Impact on Muscle Weakness of Various IV Ketogenic Formulae in PF Treated Septic Mice (Study 2)

**FIGURE 2:**
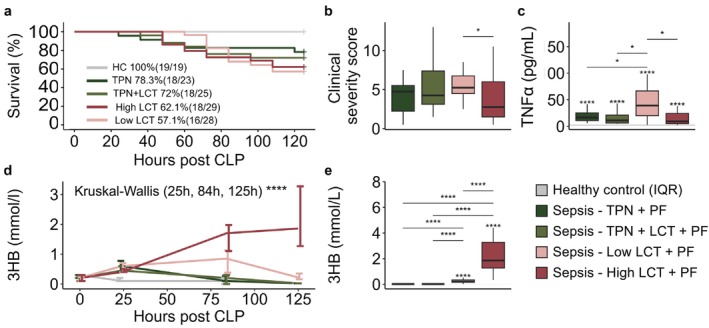
Impact of pharmacological PPARα activation and different macronutrient infusions on survival, severity of illness and ketosis. (a) Survival after 5 days of sepsis. (b) Cumulative pain score of surviving septic mice. (c) Plasma concentrations of tumour necrosis factor α. (d) Blood concentrations of 3‐hydroxybutyrate (3HB). (e) Plasma concentrations of 3HB. Healthy mice *n* = 19; TPN + PF *n* = 18; TPN + LCT + PF *n* = 18; Low‐LCT + PF *n* = 16; High‐LCT + PF *n* = 18. Abbreviations: CLP, caecal ligation and puncture; TNFα, tumour necrosis factor alpha. The interquartile range of HC mice is shown in grey, and asterisks above boxplots denote comparisons with HC mice. Statistical significance is shown by */**/***/****: *p* < 0.05/0.01/0.001/0.0001. In the line plot, overall statistical difference was assessed by the Kruskal–Wallis test for each time point (0, 25, 84 and 125 h). Illustrations were created with BioRender.com.

To further investigate whether PF treatment would be able to induce ketosis and attenuate muscle weakness in a ketogenic nutritional context, we next compared PF treatment in a group receiving standard balanced‐TPN, a group receiving TPN + LCT, a group receiving pure LCT in a low dose and a group receiving pure LCT in a high dose. After 5 days of sepsis, overall mortality was not different among the septic groups (*p* = 0.5), although survival tended to be lower for the two groups receiving the pure LCT emulsions (Figure 2a). In the Low‐LCT group, clinical illness severity scores tended to be higher and TNFα plasma concentrations were increased relative to the other septic mice (Figure 2b,c).

PF treatment in combination with balanced‐TPN did not induce detectable plasma ketosis (0.02 mmol/L), confirming the findings of the first experiment, and provision of extra LCT still did not induce ketosis (Figure 2d). Conversely, 3HB levels rose significantly in the two pure‐LCT groups (> 10‐fold increase), which had not received carbohydrates or proteins, with the highest increment in the High‐LCT group (95‐fold increase) (Figure 2d,e).

### 3.3 Impact of Various IV Ketogenic Formulae on Markers of Muscle and Liver Function in PF Treated Septic Mice

**FIGURE 3:**
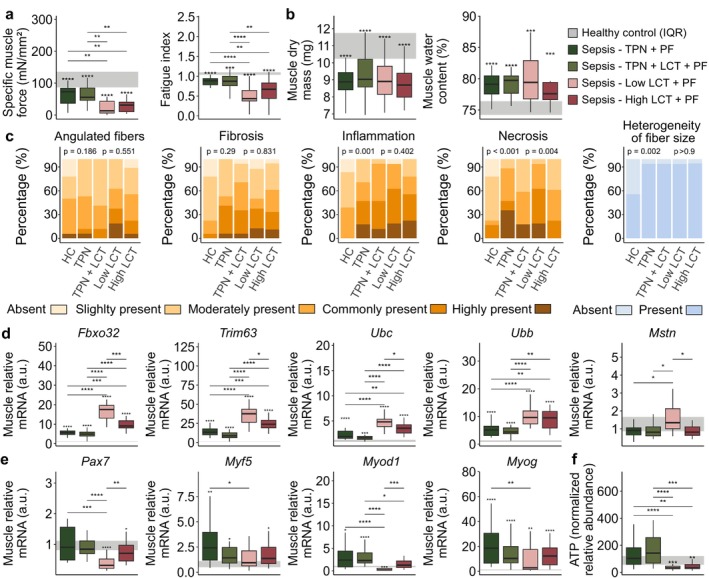
Impact on muscle function and integrity. (a) Specific maximal muscle force and muscle fatigue index. (b) Dry mass of EDL muscle and muscle water content. (c) Muscle histological analysis by H&E staining estimating the presence of angulated fibres, fibrosis, inflammation, necrosis and the heterogeneity of fibre size. (d) Relative mRNA expression of genes involved in the ubiquitin‐proteasome system in muscle tissue. (e) Relative mRNA expression of genes involved in muscle regeneration. (f) Muscle tissue levels of ATP. Abbreviations: a.u., arbitrary unit; EDL, extensor digitorum longus; rel., relative. The interquartile range of HC mice is shown in grey, and asterisks above boxplots denote comparisons with HC mice. Statistical significance is shown by */**/***/****: *p* < 0.05/0.01/0.001/0.0001. For histological analyses, left‐sided *p*‐values denote overall statistical comparison assessed by the Kruskal–Wallis test among all groups and right‐sided *p*‐values among the septic mice.

**FIGURE 4:**
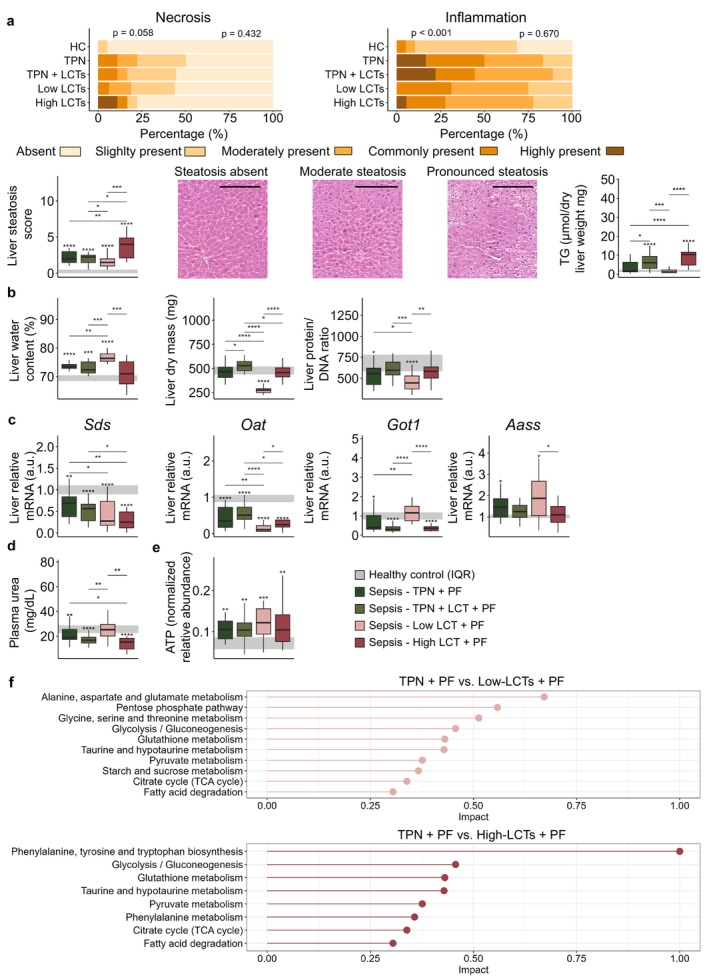
Impact on hepatic integrity and amino acid metabolism. (a) Histological analysis of signs of hepatic necrosis, inflammation and lipid accumulation. (b) Water content, dry mass and protein to DNA ratio of liver tissue. (c) Relative mRNA expression of enzymes involved in proteolysis. (d) Plasma urea levels after 5 days of sepsis. (e) ATP content of liver tissue. (f) Representation of pathways most enriched in the Low‐LCT and High‐LCT groups, resp., relative to the TPN group. Abbreviations: a.u., arbitrary unit; DAG, diacylglycerol; TAG, triacylglycerol. The interquartile range of HC mice is shown in grey, and asterisks above boxplots denote comparisons with HC mice. Statistical significance is shown by */**/***/****: *p* < 0.05/0.01/0.001/0.0001. Black scale bars in histological images denote 200 μm. For histological analyses, left‐sided *p*‐values denoted overall statistical comparison assessed by the Kruskal–Wallis test among all groups and right‐sided *p*‐values among the septic mice. Illustrations were created with BioRender.com.

Septic mice receiving PF combined with balanced TPN had lower muscle force than healthy control mice (41.6% decline in median muscle force relative to HC mice, *p* < 0.0001; Figure 3a), and adding extra LCTs to the TPN did not attenuate sepsis‐induced muscle weakness (65.4% decline in median muscle force relative to HC mice, *p* < 0.0001; Figure 3a). Remarkably, in both groups receiving PF combined with pure LCTs, both muscle strength and muscle endurance declined further despite increased ketosis (89.3% and 75.0% decline in median muscle force for Low‐LCT and High‐LCT groups relative to HC mice, resp., *p* < 0.0001; Figure 3a). This substantial decline in muscle function contrasted with the relative maintenance of muscle mass and structure. Indeed, all septic groups similarly revealed sepsis‐induced loss of muscle mass and muscle oedema (Figure 3b). Also, the number of angulated fibres, presence of fibrosis and inflammation, presence of necrosis and heterogeneity in fibre size were mostly similar among all septic groups on histological assessment (Figure 3c) as were the percentage of rounded fibres and internalised nuclei (Supplementary Figure S3). However, the sepsis‐induced upregulation of atrophy markers (*Fbxo32*, *Trim63*, *Ubc*, *Ubb*) was further increased in the Low‐LCT and High‐LCT groups, while expression of myostatin (*Mstn*) was higher only in the Low‐LCT group (Figure 3d). Additionally, markers of muscle regeneration (*Pax7*, *Myf5*, *Myod1* and *Myog*) were overall lower in both pure LCT groups, but especially in the Low‐LCT group (Figure 3e). Also, direct metabolic changes might have been important contributing factors to the aggravation of muscle weakness in the pure LCT groups. Indeed, metabolomic analyses revealed that oxidative processes were unable to maintain muscle adenosine triphosphate (ATP) levels in septic mice receiving PF with the pure LCT emulsion resulting in a muscle bioenergetic failure (Figure 3f). In contrast to muscle function, markers of liver function and integrity were similarly affected among the septic groups, with similar plasma γGT, hepatic gene expression markers of inflammation (*Tnf*, *Il6*, *Il1b* and *Nlrp3*) and histological signs of necrosis and inflammation (Figure 4a and Supplementary Figure S4). The high doses of lipids in the High‐LCT group did, however, result in hepatosteatosis (Figure 4a). The sepsis‐induced liver oedema was most pronounced in the Low‐LCT group, in which liver dry mass and liver protein/DNA ratio were also markedly decreased (Figure 4b). Overall, protein catabolism appeared to be further aggravated in the Low‐LCT group, as hepatic markers of catabolic enzymes were altered relative to the other septic mice, and plasma urea levels were elevated (Figure 4c,d). Contrary to the Low‐LCT group, urea in the High‐LCT group tended to be similar to the two TPN groups. Nonetheless, as no amino acids were administered in the High‐LCT group, their plasma urea levels were generated exclusively by endogenous ureagenesis, suggesting also increased amino acid catabolism in the High‐LCT group. In line with these findings, pathway analysis of liver metabolomics revealed enriched amino acids pathways in both pure LCT groups relative to the TPN groups (Figure 4f). In contrast with muscle tissue, liver ATP levels were maintained in all septic groups (Figure 4e).

The unanticipated metabolic failure in muscle tissue with pure lipid administration prompted additional metabolic analyses to investigate the relative contribution of lipids and glucose to oxidative processes in liver and muscle tissue.

### 3.4 Impact of Various IV Ketogenic Formulae on Markers of Lipid Metabolism in Liver and Muscle Tissue in PF Treated Septic Mice

**FIGURE 5:**
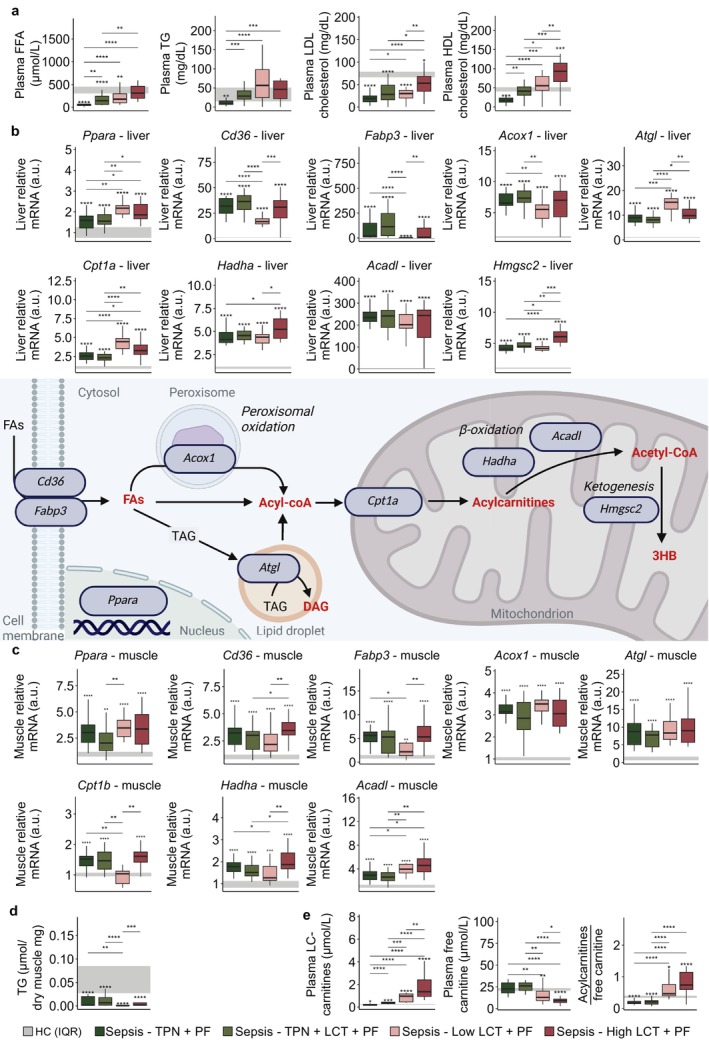
Impact on hepatic and muscle lipid metabolism. (a) Plasma lipid profile (FFA, TG, LDL and HDL) after 5 days of sepsis. Relative mRNA expression of PPARα and its downstream genes in (b) liver and (c) muscle tissue. (d) Triglyceride content of muscle tissue. (e) Plasma long‐chain and free carnitine concentrations, and the ratio of the sum of acylcarnitines to free carnitine, resp. Abbreviations: a.u., arbitrary unit; DAG, diacylglycerol; TAG, triacylglycerol. The interquartile range of HC mice is shown in grey, and asterisks above boxplots denote comparisons with HC mice. Statistical significance is shown by */**/***/****: *p* < 0.05/0.01/0.001/0.0001. Illustrations were created with BioRender.com.

The impact of the intervention on lipid metabolism was first investigated as lipids were the main substrate available for oxidation. The reduction in plasma lipid levels (FFAs, TGs, LDL‐ and HDL‐cholesterol) observed in the balanced‐TPN group as compared with healthy controls was counteracted in the TPN + LCT, Low‐LCT and mostly so in the High‐LCT group (Figure 5a). Relative to HC mice, all PF treated septic mice had upregulation of expression of PPARα (*Ppara*) and its other downstream targets involved in lipolysis, beta‐oxidation and ketogenesis in all septic groups in liver and muscle tissue, with an overall further increase in hepatic *Ppara* in the pure LCT groups (Figure 5b,c).

Despite the increased lipid availability in the intravascular compartment in the two pure LCT groups, relative abundances of FFAs in muscle tissue remained unaffected by the intervention (Supplementary Figure S5a–c), and overall TG muscle content was decreased in all septic groups, mostly so in the Low‐LCT group (Figure 5d). Conversely, in liver tissue, provision of extra LCT (TPN + LCT) or pure LCTs increased FFA content (Supplementary Figure S5d–f). Expression of hepatic and muscle markers of lipid transport (*Cd36* and *Fabp3*) was relatively downregulated in the Low‐LCT group, despite being targets of PPARα (Figure 5b,c). In addition, plasma long‐chain acylcarnitines were substantially elevated in the two pure LCT groups, while plasma free carnitine was markedly lower, especially in the High‐LCT group (Figure 5e). This resulted in a higher ratio of acylated carnitines to free carnitine (Figure 5e), potentially suggesting decreased oxidative capacity [28].

**FIGURE 6:**
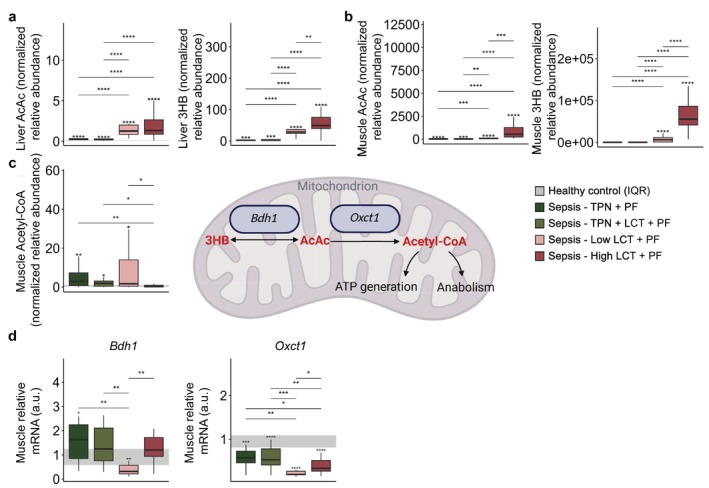
Impact on ketone body metabolism in liver and muscle tissue. (a) Acetoacetate and β‐Hydroxybutyric acid levels in liver tissue. (b) Acetoacetate and β‐jydroxybutyric acid levels in muscle tissue. (c) Acetyl‐CoA in muscle tissue. (d) Relative mRNA expression of rate‐limiting enzymes of ketolysis (Bdh1 and Oxct1). Abbreviations: a.u., arbitrary unit. The interquartile range of HC mice is shown in grey, and asterisks above boxplots denote comparisons with HC mice. Statistical significance is shown by */**/***/****: *p* < 0.05/0.01/0.001/0.0001. Illustrations were created with BioRender.com.

Parallel to the plasma profile, ketone bodies 3HB and acetoacetate (AcAc) levels were significantly higher in the two pure LCT groups in both liver and muscle tissue (Figure 6a,b). However, despite the high availability of ketone bodies in the two pure LCT groups, muscle acetyl‐CoA levels were not substantially elevated (Figure 6c). Moreover, mRNA expression of the enzyme responsible for the conversion between 3HB and AcAc (*Bdh1*) was reduced in the Low‐LCT group and the flux‐regulating enzyme of ketolysis (*Oxct1*) was suppressed in both pure LCT groups, further implying impaired ketolytic capacity (Figure 6d).

### 3.5 Impact of Various IV Ketogenic Formulae, as Compared With Balanced‐TPN, on Markers of Glucose Metabolism in PF Treated Septic Mice

#### 3.5.1 Hepatic Gluconeogenesis

**FIGURE 7:**
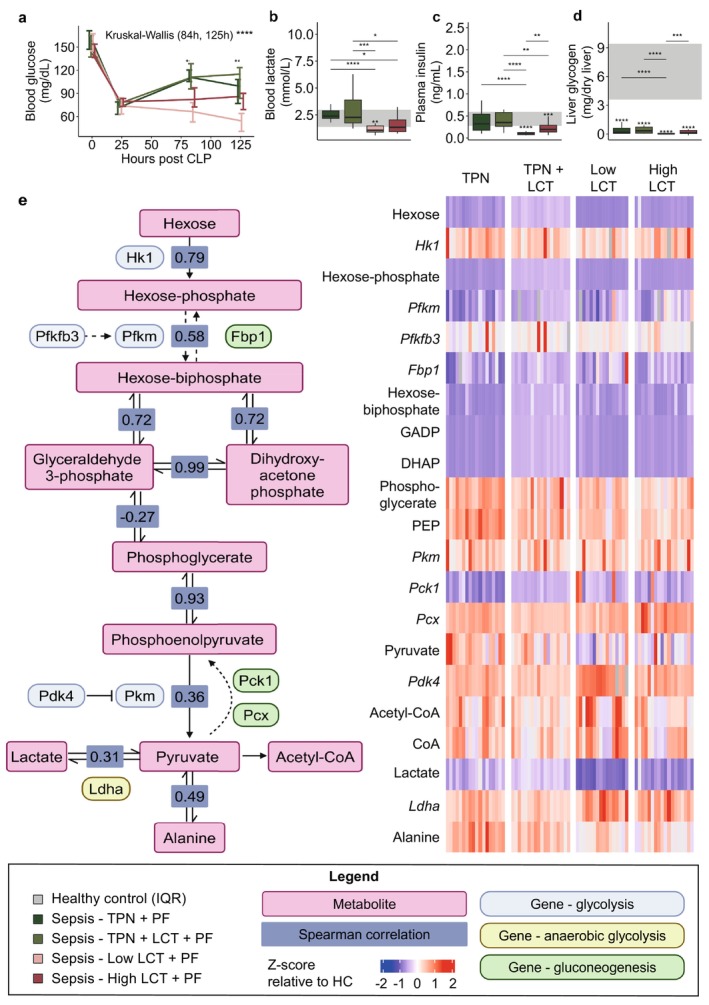
Impact on hepatic glucose metabolism and gluconeogenesis. (a) Blood glucose levels throughout the 5‐day study period. (b) Blood lactate levels. (c) Plasma insulin levels. (d) Hepatic glycogen content. (e) Heatmap of metabolites and mRNA expression genes encoding for rate‐limiting enzymes of glycolysis and gluconeogenesis in liver tissue. Statistical comparisons can be found in the supplemental information (Supplementary Table S5). Abbreviations: DHAP, dihydroxy‐acetone phosphate; GADP, glyceraldehyde 3‐phosphate; PEP, phosphoenolpyruvate; TCA, tricarboxylic acid. The interquartile range of HC mice is shown in grey, and asterisks above boxplots denote comparisons with HC mice. Statistical significance is shown by */**/***/****: *p* < 0.05/0.01/0.001/0.0001. In the line plot, the overall statistical difference among septic mice was assessed by the Kruskal–Wallis test for each time point (0, 25, 84 and 125 h). Illustrations were created with BioRender.com.

As glucose was not administered in the two pure LCT groups, glycemia depended entirely on endogenous gluconeogenesis in these groups. Blood glucose levels, plasma lactate and insulin levels were decreased in the two pure LCT groups as compared with the other septic groups (Figure 7a–c). This occurred despite upregulated expression of hepatic markers of gluconeogenesis (*Pck1 and Pcx*) (Figure 7e), while hepatic glycogen and glycolytic liver intermediates Hexose(P), GADP and DHAP were depleted in all septic groups, suggesting exhausted hepatic glucose metabolism (Figure 7d,e).

Remarkably, despite these upstream changes, hepatic phosphoglycerate, phosphoenolpyruvate (PEP) and pyruvate and acetyl‐CoA levels remained high to normal in all septic groups (Figure 7e). Correlation analysis showed that the relative abundances of pyruvate correlated moderately with phosphoenolpyruvate (Spearman correlation: 0.36, *p* = 0.0008), and more strongly with alanine (Spearman correlation: 0.49, *p* = 0.006) (Figure 7e). Also, the Cori cycle, which converts lactate to pyruvate, appeared to be upregulated in the two pure LCT groups as both plasma and hepatic lactate levels were lower in both groups, and lactate dehydrogenase (*Ldha*) mRNA levels were slightly higher in the Low‐LCT group (Figure 7b–e). Pathway enrichment analysis supported significantly altered gluconeogenesis and pyruvate metabolism in the two pure LCT groups relative to the TPN groups (Figure 4f). Overall, these results suggest that hepatic gluconeogenesis was activated but could not maintain adequate glucose homeostasis.

#### 3.5.2 Muscle Glucose Oxidative Metabolism, the Tricarboxylic Acid Cycle (TCA) and the Oxidative Phosphorylation

**FIGURE 8:**
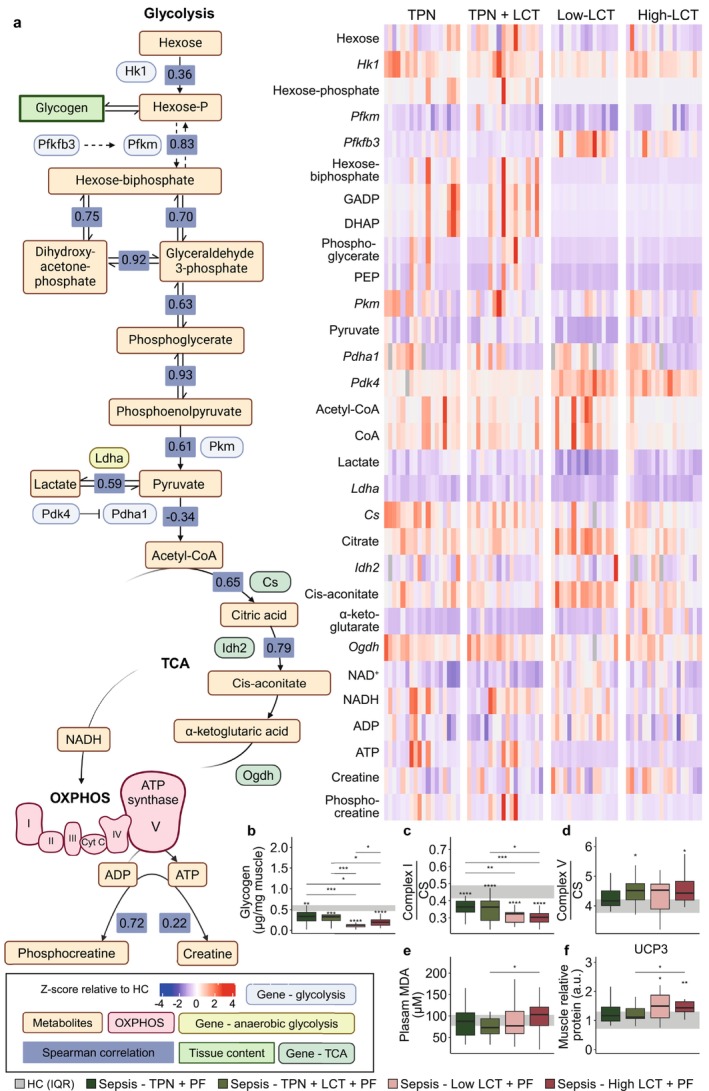
Impact on muscle oxidative metabolism, mitochondrial function and bioenergetic profile. (a) Heatmap of muscle metabolites and mRNA expression of genes encoding for rate‐limiting enzymes involved in glycolysis, pyruvate oxidation, the initial steps of the tricarboxylic acid (TCA) cycle and the ATP buffering system. Statistical comparisons can be found in the supplemental information (Supplementary Table S6). (b) Glycogen content in muscle tissue. Mitochondrial activity of complex I (c) and complex V (d) relative to the activity of citrate synthase. (e) Plasma MDA concentrations. (f) Muscle relative protein expression of UCP3. Abbreviations: CS, citrate synthase; DHAP, dihydroxy‐acetone phosphate; GADP, glyceraldehyde 3‐phosphate; MDA, malondialdehyde; PEP, phosphoenolpyruvate; TCA, tricarboxylic acid; UCP, uncoupling protein. The interquartile range of HC mice is shown in grey, and asterisks above boxplots denote comparisons with HC mice. Statistical significance is shown by */**/***/****: *p* < 0.05/0.01/0.001/0.0001. Illustrations were created with BioRender.com.

In parallel with plasma and liver tissue, relative abundances of hexose (P) and glycogen in muscle tissue were decreased in the pure LCT groups (Figure 8a,b). Expression of rate‐limiting glycolytic enzymes showed divergent signals (overall increased expression of *Hk1*, decrease in *Pfkm* and no change in *Pkm* in all septic mice) (Figure 8a), but expression of *Pfkfb3*, which is a potent stimulator of glycolysis, was upregulated in the pure LCT groups (Figure 8a). Muscle glycolytic intermediates (hexose‐phosphate, hexose‐biphosphate, GADP, DHAP, phosphoglycerate, phosphoenolpyruvic acid) were markedly lower in the pure LCT groups than in the other septic mice, but pyruvate levels were significantly lower in the Low‐LCT group only (Figure 8a). Similar to the hepatic values, muscle lactate levels were lower in both pure LCT groups, while *Ldha* gene expression was further suppressed (Figure 8a). Muscle mRNA expression of pyruvate dehydrogenase (*Pdha1*) was similar between groups, but its enzymatic inhibitor (pyruvate dehydrogenase kinase, *Pdk4*) was elevated in all groups, most pronounced in the pure LCT groups (Figure 8a). In correlation analysis, pyruvate strongly correlated with muscle lactate (Spearman correlation: 0.59, *p* < 0.0001) and PEP (Spearman correlation: 0.61, *p* < 0.0001), while there was a relatively low and inverse correlation with acetyl‐CoA levels (Spearman correlation: −0.34, *p* < 0.0001) (Figure 8a). Together, these findings support substantial alterations in muscle glycolytic pathways in the pure LCT groups.

Despite high lipid dose provision, muscle acetyl‐CoA and free CoA levels remained relatively low in the pure LCT groups, and acetyl‐CoA appeared to be closely related to citrate levels (Spearman correlation: 0.65, *p* < 0.0001) (Figure 8a). As expression levels of key TCA cycle enzymes were decreased in the Low‐LCT group (Figure 8a), deceleration of the TCA cycle in this group cannot be excluded. Conversely, except for an increase in citrate and cis‐aconitate in the Low‐LCT group and α‐ketoglutarate in the High‐LCT group in comparison with the other septic mice (Figure 8a), TCA metabolites were overall similar among the groups.

In muscle, the activity of complex I of the oxidative phosphorylation was lower in the two pure LCT groups than in the other septic mice, while the function of complex V appeared to be overall relatively unaffected (Figure 8c,d). Plasma malondialdehyde (MDA), a marker of lipid peroxidation and oxidative stress [29], was only slightly elevated in the High‐LCT group (Figure 8e). Additionally, relative protein expression of uncoupling protein 3 (UCP3) in muscle tissue tended to be higher in both pure LCT groups (Figure 8f). Given the alterations in glycolysis, pyruvate, acetyl‐CoA and TCA cycle intermediates, the cause of the low ATP levels, and thus of the muscle bioenergetic failure, appeared to originate mostly from impaired metabolic substrate delivery to the OXPHOS system, rather than structural mitochondrial dysfunction. Additionally, ATP‐buffering systems were affected as shown by the higher ADP and lower phosphocreatine levels in the two pure LCT groups (Figure 8a).

## 4 Discussion

In this study of septic mice, pharmacological activation of PPARα combined with balanced‐TPN infusion did not induce ketosis nor affect muscle weakness, irrespective of the amount of LCT provided. In the absence of carbohydrates and amino acids, however, PPARα activation combined with LCT infusion resulted in a stable ketosis, but surprisingly, further aggravated rather than improved muscle weakness possibly explained by the induction of bioenergetic failure.

Pharmacological activation of PPARα with PF appeared safe and well‐tolerated in our standardised murine model of prolonged sepsis, as mortality and illness severity were seemingly unaffected. This contrasts with previous studies of acute sepsis, where PPARα agonism was reported to reduce mortality and inflammation in rodent sepsis [12, 30]. However, in these acute models, supportive care with fluid resuscitation and antibiotic treatment was minimal, and the treatment was given prior to the onset of sepsis rendering comparison difficult and questioning the clinical validity [12, 30]. Moreover, the metabolic impact of PF treatment in these studies was not investigated in detail. In the first experiment of the current study, the metabolic impact during prolonged sepsis and concomitant balanced‐TPN infusion appeared to be overall limited and failed to induce ketosis, even when the infusion of LCTs was increased. Indeed, the high glucose load in the TPN infusion led to relatively high insulin plasma concentrations, likely suppressing ketogenesis, which could not be overcome by PF treatment. Unexpectedly, PF treatment with pure LCT infusion, in the absence of carbohydrates and amino acids, inflicted harm instead of benefit. This distinct phenotype was marked by ketosis, but also by steatosis complications, lower survival trends, increased inflammation and a notable exacerbation in muscle weakness. The decline in muscle function in both pure LCT groups appeared to be mostly of metabolic and functional origin as muscle mass and muscle structure on histological analysis were not further affected, while muscle ATP levels were much depleted in these groups. Abnormalities of mitochondrial ultrastructure cannot be excluded [31]. This myofibre metabolic failure was likely caused by an interplay of multiple interconnected factors. LCTs require carnitine for intramitochondrial shuttling and their beta‐oxidation is more complex than for other FFAs with a shorter chain length [32, 33]. This may have induced a beta‐oxidative bottleneck due to a mismatch between lipid load and oxidative capacity, particularly under conditions such as extreme ketogenic diets with limited glucose and amino acid availability. Indeed, long‐chain carnitine levels were extremely elevated in both pure LCT groups, while muscle acetyl‐CoA levels remained relatively low. Moreover, plasma MDA concentrations were only marginally elevated during high LCT provision, and muscle UCP3 only tended to be higher in both pure LCT groups, arguing against a marked augmentation of lipid (per)oxidation or oxidative stress. Additionally, enhanced lipid and ketone body metabolism may have sequestered free CoA to generate fatty acyl‐CoA groups. Plasma free carnitine levels were indeed decreased in the two pure LCT groups, while acylcarnitines accumulated, suggesting enhanced buffering of fatty‐acyl groups by carnitine, a mechanism potentially contributing to maintaining CoA availability. Furthermore, insufficient CoA availability may have impeded proper functioning of the TCA cycle and other metabolic oxidative processes.

Nevertheless, theoretically, metabolic pathways should be capable of providing sufficient energy from a pure LCT emulsion through indirect and direct generation of acetyl‐CoA by ketone bodies and FFAs, respectively. Previous studies have shown that a zero‐carbohydrate, High‐LCT diet in C57bl6 mice led to insulin resistance and weight gain, but without severe complications such as premature death or (severe) muscle weakness [34, 35]. Of note, these study diets included a small proportion of amino acids (< 10–20%) and mice were not critically ill, altering the metabolic context of these findings. In our setup, the complete absence of exogenous glucose and amino acids in the two pure LCT groups may have prevented a fully functional TCA cycle by impaired replenishment of critical intermediates and CoA depletion, constraining acetyl‐CoA oxidation. Anaplerosis in this context was entirely dependent on glucose and amino acid generation from endogenous stores (i.e., lean body mass). PPARα agonists are postulated to also activate gluconeogenesis and affect amino acid metabolism [36], but in this context of sepsis‐induced critical illness, (hepatic) gluconeogenesis was unable to maintain glucose homeostasis as reflected by the lower glycemia and depletion of myofibre glycolytic intermediates, as well as the enrichment of gluconeogenesis pathways in the two pure LCT groups. Moreover, amino acid catabolism was enhanced and led to excessive production of nitrogenous waste in the form of urea, imposing additional metabolic stress.

Remarkably, muscle mRNA expression levels of Oxct1, which encodes the rate‐limiting enzyme of ketolysis (succinyl‐CoA 3‐oxoacid CoA‐transferase 1, SCOT), were significantly suppressed in the two pure LCT groups despite ketosis. This result ties well with previous studies wherein downregulation of Oxct1 and decreased activity of SCOT were observed during a state of prolonged ketosis [37, 38]. Moreover, perfusion of hearts by AcAc decreased myocardial function and impaired TCA flux, while supplementation (of synthetic precursors) of CoA or anaplerotic substrates resolved this defect [39, 40]. One might speculate that the downregulation of Oxct1 during ketosis might function as a feedback mechanism to prevent consumption of CoA during ketolysis and might serve as a protective mechanism to preserve CoA availability [39, 40]. In that respect, the relatively high levels of plasma 3HB might have also been the result of ketone bodies accumulating by decreased ketolysis and oxidation. Collectively, these disruptions underscore the intricate interplay between macronutrient availability, hormonal regulation and metabolic flexibility, which may have resulted in impaired substrate oxidation and eventually bioenergetic failure. The relation between Oxct1/SCOT and CoA availability should be further addressed in other studies.

In contrast with our current findings, in previous studies examining direct parenteral administration of 3HB or different ketogenic PN, induction of ketosis during sepsis was almost exclusively associated with improved muscle function [4, 41]. The results in this study emphasise the importance of the metabolic context to determine whether ketone bodies may mediate improved muscle functionality and overall outcome or rather act as a symptom of metabolic dysfunction. As such, our results should also caution against aggressive ketogenic nutritional strategies in critically ill patients without provision of carbohydrates or amino acids. To this end, it seems prudent that the additional glucose and/or amino acids should be sufficiently high to enhance the potentially impaired glycolytic and anaplerotic pathways, while preventing suppression of ketogenesis. Commonly used ketogenic diets typically limit glucose intake to less than 10% of the total caloric intake [42]. Likewise, high dosages of propofol, an anaesthetic commonly dissolved in a LCT emulsion and with an inherent risk to mitochondrial uncoupling (i.e., propofol infusion syndrome), have been associated with detrimental metabolic consequences [43, 44, 45]. Critically ill patients receiving high doses of such a lipid‐based drug vehicle might thus potentially be at risk for developing metabolic complications with a detrimental impact on muscle function, which should be further addressed in additional studies.

This study has several limitations. Assessment of metabolic function and parameters was mostly conducted by point‐estimate analyses instead of tracer experiments, rendering the actual activity of pathways uncertain. Since we first tested PF treatment in mice receiving balanced‐TPN and then tested various macronutrient proportions of PN in PF‐treated septic mice, it is unclear to which extent the PF administration itself may have affected metabolic pathways. This interaction needs to be further investigated. Also, the effect of PF on PPARα activation was tested on the level of target gene expression only. Nonetheless, this study revealed a distinct phenotype and clearly demonstrates the potentially harmful effects of a nutritionally or pharmacologically forced ketogenesis during prolonged sepsis. Whether the tested interventions might exert different effects in the acute, more prolonged or recovery phase of sepsis, where the metabolic context is likely different, should be further addressed in other experimental designs. It remains unclear if in the presence of minimal amounts of glucose or amino acids, PPARα activation in combination with a high‐dose lipid PN might attenuate muscle weakness and be safe from a metabolic perspective. Moreover, the metabolic interventions tested in the current study were only assessed in male mice, in whom abdominal sepsis was induced by a CLP. Whether female mice might develop a similar phenotype or whether other aetiologies of sepsis may affect the metabolic response remains to be determined.

To conclude, in septic mice, PF‐induced PPARα activation combined with balanced‐TPN was unable to induce ketosis and did not attenuate muscle weakness. Unexpectedly, in septic mice, PF combined with pure LCT PN, as compared with PF with balanced‐TPN or lipid‐rich TPN, resulted in ketosis, but aggravated rather than improved muscle weakness, irrespective of the LCT dose. In these pure LCT groups, metabolic analyses suggested a bioenergetic failure of muscle fibres with an accumulation of plasma lipids and with high‐LCT also the development of liver steatosis.

## Ethics Statement

The Institutional Ethical Committee for Animal Experimentation of the KU Leuven had approved the protocols for these animal studies (project numbers P180/2016 and P097/2021). All experiments were performed in accordance with the ethical standard laid down in the 1964 Declaration of Helsinki and its later amendments. The manuscript does not contain clinical studies or patient data.

## Conflicts of Interest

The authors declare no conflicts of interest.

## Supporting information


**Table S1:** Missingness per randomisation group for each analysis for Study 1.
**Table S2:** Missingness per randomisation group for each analysis for Study 2.
**Table S3:** Composition of parenteral nutrition per septic group.
**Table S4:** List of TaqMan gene expression assays.
**Table S5:** Univariate statistical comparisons among groups for metabolites and mRNA levels shown in the heatmap of Figure 7 in liver tissue.
**Table S6:** Univariate statistical comparisons among groups for metabolites and mRNA levels shown in the heatmap of Figure 8 in muscle tissue.
**Figure S1:** Schematic overview of study design and surviving animals in Studies 1 and 2. Abbreviations: IV, intravenous; LCT, long‐chain triglyceride; PF, pemafibrate; TPN, total parenteral nutrition.
**Figure S2:** Housekeeping genes per cDNA batch for liver tissue (Rn18s, panel A) in Study 1 and gastrocnemius (SDHA, panels B–D) and liver tissue (Rn18s, panels E‐F) for Study 2, real‐time PCR analyses. Abbreviations: cDNA, copy deoxyribonucleic acid; CT, cycle threshold; IV, intravenous; LCT, long‐chain triglyceride; PF, pemafibrate; TPN, total parenteral nutrition. Statistical significance is shown by */**/***/****: p < 0.05/0.01/0.001/0.0001. Asterisks placed above boxplots denote comparisons with HCs, and the interquartile ranges of HC mice is shown in grey.
**Figure S3:** Muscle histological analysis by H&E staining, including (A) the number of rounded fibres and (B) internalised nuclei. Representative muscle histology images are shown per randomisation group, and black scale bars denote 200 μm. The interquartile range of HC mice is shown in grey.
**Figure S4:** Markers of liver function, cytokine levels and histological integrity. (A) Plasma γ‐GT levels after 5 days of sepsis. Relative mRNA expression of liver cytokines, including (B) TNF, (C) Il‐6, (D) Il‐1b and (E) Nlrp3. (F) Representative liver histology images are shown per randomisation group, and black scale bars denote 200 μm. Statistical significance is shown by */**/***/****: p < 0.05/0.01/0.001/0.0001. Asterisks placed above boxplots denote comparisons with HCs, and the interquartile ranges of HC mice is shown in grey. Abbreviations: γ‐GT: γ‐glutamyltransferase.
**Figure S5:** Free fatty acids including palmitic acid, stearic acid and myristic acid in muscle (panels A, B and C, respectively) and liver tissue (panel D, E and F, resp.). The interquartile range of HC mice is shown in grey. Statistical significance is shown by */**/***/****: p < 0.05/0.01/0.001/0.0001. Asterisks placed above boxplots denote comparisons with HCs.
