# Supplementary material for: Ketogenic Nutrition in Combination With PPARα Activation Induced Metabolic Failure and Exacerbated Muscle Weakness in Septic Mice
Source: J Cachexia Sarcopenia Muscle. 2025 Dec 9;16(6):e70156. doi: 10.1002/jcsm.70156 (PMC12688406; doi:10.1002/jcsm.70156)
Supplement: Supplementary file 1 — Table S1: Missingness per randomisation group for each analysis for Study 1. Table S2: Missingness per randomisation group for each analysis for Study 2. Table S3: Composition of parenteral nutrition per septic group. Table S4: List of TaqMan gene expression assays. Table S5: Univariate statistical comparisons among groups for metabolites and mRNA levels shown in the heatmap of Figure 7 in liver tissue. Table S6: Univariate statistical comparisons among groups for metabolites and mRNA levels shown in the heatmap of Figure 8 in muscle tissue. Figure S1: Schematic overview of study design and surviving animals in Studies 1 and 2. Abbreviations: IV, intravenous; LCT, long‐chain triglyceride; PF, pemafibrate; TPN, total parenteral nutrition. Figure S2: Housekeeping genes per cDNA batch for liver tissue (Rn18s, panel A) in Study 1 and gastrocnemius (SDHA, panels B–D) and liver tissue (Rn18s, panels E‐F) for Study 2, real‐time PCR analyses. Abbreviations: cDNA, copy deoxyribonucleic acid; CT, cycle threshold; IV, intravenous; LCT, long‐chain triglyceride; PF, pemafibrate; TPN, total parenteral nutrition. Statistical significance is shown by */**/***/****: p < 0.05/0.01/0.001/0.0001. Asterisks placed above boxplots denote comparisons with HCs, and the interquartile ranges of HC mice is shown in grey. Figure S3: Muscle histological analysis by H&E staining, including (A) the number of rounded fibres and (B) internalised nuclei. Representative muscle histology images are shown per randomisation group, and black scale bars denote 200 μm. The interquartile range of HC mice is shown in grey. Figure S4: Markers of liver function, cytokine levels and histological integrity. (A) Plasma γ‐GT levels after 5 days of sepsis. Relative mRNA expression of liver cytokines, including (B) TNF, (C) Il‐6, (D) Il‐1b and (E) Nlrp3. (F) Representative liver histology images are shown per randomisation group, and black scale bars denote 200 μm. Statistical significance is shown by */**/***/****: [file JCSM-16-e70156-s001.pdf]

Ketogenic Nutrition in Combination with PPARα Activation Induced Metabolic Failure and Exacerbated Muscle Weakness in Septic Mice

Caroline Lauwers[1], Wouter Vankrunkelsven[1], Sarah Derde[1], Sarah Vander Perre [1], Inge Derese[1], Lies Pauwels[1], Ilse Vanhorebeek[1], Louis Libbrecht[2], Pieter Vermeersch[3], Jan Gunst[1], Greet Van den Berghe[1], Michael P. Casaer[1], Lies Langouche[1]

1 Department of Cellular and Molecular Medicine, Laboratory and Clinical division of Intensive Care Medicine, KU Leuven, Leuven, Belgium.

2 Laboratory of Hepatology, KU Leuven, Leuven, Belgium.

3 Clinical department of Laboratory Medicine, UZ Leuven, Leuven, Belgium.

ORCID IDs:
Caroline Lauwers: 0000-0001-7278-2708
Wouter Vankrunkelsven: 0000-0003-0943-043X
Sarah Derde: 0009-0005-6724-643X
Ilse Vanhorebeek: 0000-0002-5261-5192
Louis Libbrecht: 0000-0002-9453-3355
Pieter Vermeersch: 0000-0001-7076-061X
Jan Gunst: 0000-0003-2470-6393
Greet Van den Berghe: 0000-0002-5320-1362
Michael P. Casaer: 0000-0002-7087-0795
Lies Langouche: 0000-0002-8564-6809

Corresponding author: prof. Lies Langouche, lies.langouche@kuleuven.be, +32 16 33 05 24

Supplemental information

TABLE OF CONTENTS

Supplemental information 2
Material and methods 3
Animal studies 3
Histological analyses 3
Metabolomics analyses 5
Supplementary Tables 6
Supplementary Table S1: missingness per randomization group for each analysis for study 1 6
Supplementary Table S2: missingness per randomization group for each analysis for study 2 7
Supplementary Table S3: composition of parenteral nutrition per septic group 9
Supplementary Table S4: list of TaqMan gene expression assays 10
Supplementary Table S5: univariate statistical comparisons among groups for metabolites and mRNA levels shown in the heatmap of Fig. 7 in liver tissue 12
Supplementary Table S6: univariate statistical comparisons among groups for metabolites and mRNA levels shown in the heatmap of Fig. 8 in muscle tissue 14
Abbreviations: GADP: Glyceraldehyde 3-phosphate; DHAP: Dihydroxy-acetone phosphate; PEP: Phosphoenolpyruvate. 15
Supplementary Figures 16
Supplementary Fig. S1 16
Supplementary Fig. S2 17
Supplementary Fig. S3 18
Supplementary Fig. S4 19
Supplementary Fig. S5 20
References 21

Material and methods

Animal studies

At the start of the experiment, mice were anesthetized, and a first subcutaneous PF was administered after which a central venous line was placed surgically in the central jugular vein. The central venous line was connected with a swivel device to allow free motion and continuous infusion. Afterwards, a median laparotomy was performed, and the caecum was ligated and puncture by a 18G needle (50% ligation and single-needle puncture through-and-through). To mimic the clinical context, sepsis was treated with antibiotics (Imipenem/Cilastin) and analgetics (Buprenorphine) twice daily. Throughout the experiment mice were individually caged in a temperature, humidity and light-controlled environment. An intravenous fluid resuscitation fluid was provided in the first 24 hours (Plasmalyte A Viaflo (Baxter®) and 6% hydroxyethyl starch in a 4/1 proportion) after which mice were randomized to receive different types of parenteral nutrition. In case of technical catheter-related issues, mice were excluded from the experiment. Healthy control mice were caged individually and received an ad libitum standard chow diet. After 5 days, septic mice and healthy control mice were anesthetized by ketamine and xylazine, and the experiment was terminated by a cardiac puncture. Each experiment was completed when all groups contained at least 15 surviving animals with a successful muscle force measurement, based on a power calculation with an estimated effect size of 1.062 (difference between means divided by a common standard deviation), $\alpha<0.05$ and $\beta>80\%$ (mean difference of 34 mN/mm^2 in specific muscle force with a common standard deviation of 32). As septic male mice show a more robust reduction in muscle force after CLP with a larger effect size than female mice, in combination with the slight overrepresentation of the male sex in critically ill patients, the cyclic hormonal influences in female mice and to comply with the 3Rs principal to minimize the number of animals in the experimental setup, the current study included only male mice.

Investigators were blinded for the randomized intervention during ex vivo muscle force measurements. After blood gas analysis, blood was collected in heparinized tubes and centrifuged. Directly post-mortem, the median liver lobe and right anterior tibialis were collected for histological assessment and the remaining liver lobes and hindleg muscles (m. gastrocnemius, m. soleus, the left m. tibialis) were snap frozen in liquid tissue for tissue analyses (rt qPCR, mitochondrial measurements, metabolomics, triglyceride and glycogen content). Plasma and tissues were stored on -80°C.

Histological analyses

Hematoxylin and eosin stained liver and muscle sections were scored by 2 independent evaluators who were blinded to the randomized intervention. In case of divergent scores, slides were reevaluated together to obtain a consensus.

Liver slides were assessed for inflammation, hypertrophy, necrosis and fat accumulation. Inflammatory infiltration was scored as: 0 (no foci per 10x field), 0.5 (few isolated inflammatory cells throughout parenchyma or <2 foci in 10x field), 1 (2-4 foci per 10x field), 1.5 (>4 foci in more than 50% of the section per 10x field) or 2 (foci in all quadrants of section per 10x field). Hepatocyte hypertrophy was assessed as 0 (absent in 10x field), 1 (regional increase in cytoplasmic volume in 10x field) or (generalized hypertrophy in 10x field). Necrosis was scored as 0 (no necrosis in 10x field), 0.5 (1 or 2 necrotic foci in 10x field), 1 (more > 2 necrotic foci in 10x field), 1.5 (necrotic areas in all quadrants in 10x field) or 2 (confluent necrosis in 10x field). Steatosis was scored by the presence of micro- and macrovesicular lipid accumulation, observed as unstained, clear circles, was scored as: 0 (<5% fat accumulation in 10x field), 0.5 (<25% fat accumulation in 10x field), 1 (25-50% fat accumulation in 10x field), 1.5 (>50% fat accumulation in 10x field) or 2 (>75% fat accumulation in 5x field). Presence of ballooning was scored as 0 (not present), 0.5 (1 or 2 per section in 10x field), 1 (>2 per section but in only 1 quadrant in 10x field), 1.5 (>3 per section in > 2 quadrants in 10x field), 2 (>5 per section in all quadrants in 10x field). The steatosis score was calculated by making a summation of the inflammation, micro- and macrovesicular lipid accumulation and ballooning hepatocyte scores [1].

Muscle sections were assessed for the presence of angulated fibers 0 (no angulated fibers), 0.5 (1-3 angulated fibers in 10x field), 1 (>3 angulated fibers limited to 1 quadrant of slide in 10x field), 1.5 (> 3 in all quadrants of slide in 10x field) or 2 (>6 in all quadrants of slide in 10x field). Fibrosis was scored as 0 (limited fibrotic tissue present in 10x field), 0.5 (slight thickening around vessels in 10x field), 1 (slight infiltration of fibrotic tissue in parenchyma in 10x field), 1.5 (severe thickening around vessels or clear fibrosis in parenchyma in 10x field), or 2 (severe thickening around vessels and clear fibrosis in parenchyma in 10x field). Necrosis was assessed as the presence of myophagocytosis (pale stained myofibers) as follows: 0 (no necrosis in 10x field), 0.5 (present in 1 quadrant in 10x field), 1 (present in > 50% of the slide in 10x field), 1.5 (present in 3 quadrants in 10x field), 2 (present in all quadrants 10x field). Inflammation was scored as: 0 (no foci per 10x field), 0.5 (slight inflammation in endomysium or perimysium in 10x field), 1 (slight inflammation in endomysium and perimysium in 10x field), 1.5 (clear increase in inflammation in the endomysium or perimysium in 10x field), 2 (clear increase in inflammation in the endomysium and perimysium in 10x field). Heterogeneity of fiber size was assessed by the absence (0) or presence of (1) of global increased heterogeneity in myofiber shape and size.

Metabolomics analyses

Muscle metabolomics data were normalized by the internal standard, log-transformed and autoscaled, and liver data were normalized by sum, cubic transformation and autoscaled.

Pathway analysis was conducted to assess global changes in pathways from metabolomic data. Pathways analysis in MetaboAnalyst integrates enrichment analysis and topological analysis from the KEGG (Kyoto Encyclopedia of Genes and Genomes) database in the MetaboAnalystR package [2].

For enrichment analysis, the global test method was used to determine which metabolic pathways were differentially affected between the 2 groups. If the p-values of the global test< 0.05, the given pathway contains significantly altered metabolites compared to a background dataset (all metabolites measurable by the metabolomics platform applied) and the pathway is significantly altered.

To assess the impact of individual metabolites within a pathway, betweenness centrality was calculated based on the KEGG (Kyoto Encyclopedia of Genes and Genomes) database. Betweenness centrality is a measure of a node's importance in network connectivity, reflecting how often a metabolite appears in the shortest paths between other metabolites and considering the global structure of the network. A higher betweenness centrality indicates a more influential metabolite in the pathway. Centrality values of metabolites were normalized by dividing each metabolite's importance by the sum of all metabolite importance scores in the pathway. This normalization ensures that the maximum pathway impact score is 1, allowing for relative comparisons between different pathways.

Supplementary Tables

Supplementary Table S1: Missingness per randomization group for each analysis for study 1

Analysis*	HC (n = 15)	Sepsis – TPN + PL (n = 15)	Sepsis – PF (n = 16)
Specific muscle force	1	0	0
Clinical severity score	0	0	2
Liver histology	0	0	2
Blood 3HB baseline	3	0	1
Blood 3HB 24h post CLP	3	1	1

* Only analyses with missingness in any of the groups are shown.

Abbreviations: HC: healthy control; TPN: total parenteral nutrition; PL: placebo; PF: pemafibrate; 3HB: 3-hydroxybutyrate; CLP: caecal ligation and puncture.

Supplementary Table S2: Missingness per randomization group for each analysis for study 2

Analysis*	HC (n = 19)	Sepsis – TPN + PF (n = 18)	Sepsis – TPN + LCT + PF (n = 18)	Sepsis – Low-LCT + PF (n = 16)	Sepsis – High-LCT + PF (n = 18)
Plasma TNFα (pg/mL)	0	1	0	0	1
Blood 3HB 84h post CLP	1	0	1	0	0
Specific muscle force	0	2	1	1	0
Fatigue index	0	2	1	2	0
Muscle dry mass (mg)	0	1	0	0	0
Muscle histology	1	0	1	1	0
Trim63/SDHA	2	3	2	1	1
Ubc/SDHA	3	0	0	0	3
Ubb/SDHA	1	2	2	2	0
Pax7/SDHA	1	1	1	0	1
Myf5/SDHA	2	3	3	0	3
Myod1/SDHA	1	3	1	0	0
Myog/SDHA	1	2	0	2	2
Sds/Rn18s	1	0	0	0	0
Got1/Rn18s	0	0	0	0	1
Aass/Rn18s	0	1	0	0	1
Plasma urea (mg/dL)	0	1	0	0	1
Plasma TG (mg/dL)	0	2	0	1	1
Plasma LDL cholesterol (mg/dL)	0	1	0	0	0
Ppara/Rn18s	0	0	1	1	0
Fabp3/Rn18s	0	1	0	0	2
Acox1/Rn18s	1	1	0	0	0
Atgl/Rn18s	2	2	1	1	1
Hadha/Rn18s	0	0	1	0	0
Acadl/Rn18s	2	1	1	1	1
Hmgcs2/Rn18s	1	1	1	0	0
Cd36/SDHA	0	0	1	0	0
Acox1/SDHA	1	1	0	0	0
Atgl/SDHA	1	0	1	0	0
Hadha/SDHA	0	1	1	0	0
Acadl/SDHA	1	0	0	0	2
Oxct1/SDHA	0	1	0	0	0
Blood glucose 84h post CLP	1	0	0	0	0

Blood glucose 125h post CLP	1	1	3	0	1
Blood lactate mmol/L)	2	4	4	0	5
Plasma insulin (ng/mL)	0	2	0	0	0
Hk1/Rn18s	0	0	0	1	1
Pfkm/Rn18s	2	0	2	1	1
Pfkfb3/Rn18s	1	0	1	0	1
Fbp1/Rn18s	1	1	0	0	0
Pdk4/Rn18s	3	0	0	2	0
Pdha1/SDHA	1	2	0	2	1
Pdk4/SDHA	1	1	0	0	0
LDHA/SDHA	1	0	0	0	0
Cs/SDHA	0	0	0	1	0
Idh2/SDHA	0	1	0	0	2
Ogdh/SDHA	0	0	0	1	0
UCP3 (relative protein expression)	1	1	1	1	1
Plasma γ-GT (IU/L)	0	2	0	1	2
Tnf/Rn18s	2	2	2	0	1
Il6/Rn18s	5	4	1	1	0
Nlrp3/Rn18s	0	2	2	0	0

* Only analyses with missingness in any of the groups are shown.

Abbreviations: HC: healthy control; TPN: total parenteral nutrition; PL: placebo; PF: pemafibrate; LCT: long-chain triglyceride; TNF: tumor necrosis factor; 3HB: 3-hydroxybutyrate; CLP: caecal ligation and puncture; TG: triglyceride; LDL: low density lipoprotein; γ-GT: γ-glutamyltransferase; UCP: uncoupling protein.

Supplementary Table S3: composition of parenteral nutrition per septic group

	Study 1		Study 2			
	TPN + PF	TPN + PF	TPN + PF	TPN + LCT + PF	Low-LCT + PF	High-LCT + PF
Total kcal provided per day	5.27	5.27	5.27	7.00	1.73	5.27
Glucose – % of total kcals per day	51.0	51.0	51.0	38.4	0	0
Amino acids – % of total kcals per day	16.2	16.2	16.2	12.2	0	0
Long-chain triglycerides – % of total kcals per day	32.8	32.8	32.8	49.4	100	100
Glucose –total kcals per day per 30g body weight*	2.69	2.69	2.69	2.69	0	0
Amino acids – total kcals per day per 30g body weight*	0.85	0.85	0.85	0.85	0	0
Long-chain triglycerides – total kcals per day per 30g body weight*	1.73	1.73	1.73	3.5	1.73	5.27
Total volume administered per day (ml)	4.8	4.8	5.76	5.76	5.76	5.76
Volume olimel N7E (ml)	4.8	4.8	4.8	4.8	0	0
Clinoleic 20% (ml)	0	0	0	0.96	0.96	2.95
Aqua ad inject (ml)	0	0	0.96	0	2.73	0.96
Plasmalyte (ml)	0	0	0	0	1.92	1.70
Electrolytes added (CaCl, KCl KPO4-, MgSO4) (ml)	0	0	0	0	0.15	0.15
Electrolytes in parenteral nutrition						
Sodium (mmol/L)	35.0	35.0	35.0	35.0	56.0	49.5
Potassium (mmol/L)	30.0	30.0	30.0	30.0	22.0	21.4
Magnesium (mmol/L)	4.0	4.0	4.0	4.0	4.0	3.9
Calcium (mmol/L)	3.5	3.5	3.5	3.5	2.5	2.5
Phosphate (mmol/L)	15.0	15.0	15.0	15.0	15.0	15.0
Acetate (mmol/L)	45.0	45.0	45.0	45.0	10.8	9.5
Gluconate (mmol/L)	0	0	0	0	9.2	8.1
Chloride (mmol/L)	45.0	45.0	45.0	45.0	44.2	49.7
Sulfate (mmol/L)	0	0	0	0	3.4	3.4

* Mice weight approximately 30g at baseline. Calculations are based on theoretical caloric composition (glucose: 1g = 4 kcal; amino acids: 1g = 4kcal; lipids: 1g = 9 kcal). Abbreviations: Pl: placebo; PF: pemafibrate; TPN: total parenteral nutrition; LCT: long-chain triglyceride; CaCl: calcium chloride; KCl: potassium chloride; KPO4-: potassium phosphate; MgSO4: magnesium sulfate.

Supplementary Table S4: list of TaqMan gene expression assays

Gene name	TaqMan Assay ID
Aass	Mm01165010_m1
Acadl	Mm00599660_m1
Acox1	Mm01246834_m1
Atgl	Mm00503040_m1
Bdh1	Mm00558330_m1
Cd36	Mm00432403_m1
Cpt1a	Mm01231183_m1
Cpt1b	Mm00487191_g1
Cs	Mm03942842_s1
Fabp3	Mm02342495_m1
Fbp1	Mm00490181_m1
Fbxo32	Mm00499523_m1
Got1	Mm01195792_g1
Hadha	Mm00805228_m1
Hk1	Mm00439344_m1
Hmgcs2	Mm00550050_m1
Idh2	Mm00612429_m1
Il6	Mm00446190_m1
Ldha	Mm01612132_g1
Mstn	Mm01254559_m1
Myf5	Mm00435125_m1
Myod1	Mm00440387_m1
Myog	Mm00446194_m1
Oat	Mm00497544_m1
Ogdh	Mm00803119_m1
Oxct1	Mm00499303_m1
Pax7	Mm01354484_m1
Pck1	Mm01247058_m1

Pcx	Mm00500992_m1
Pdha1	mm00468675_m1
Pdk4	Mm01166879_m1
Pfkfb3	Mm00504650_m1
Pfkm	Mm01309576_m1
Pkm	Mm00834102_gH
Ppara	Mm00440936_m1
Rn18s	Mm03928990_g1
Sdha	Mm01352366_m1
Sds	Mm00455131_m1
Tnf	Mm00443258_m1
Trim63	Mm01185221_m1
Ubb	Mm01622233_g1
Ubc	Mm02525934_g1

Supplementary Table S5: univariate statistical comparisons among groups for metabolites and mRNA levels shown in the heatmap of Fig. 7 in liver tissue

	P-value of Wilcoxon signed-rank test in comparison with HC animals				P-value of Wilcoxon signed-rank test in comparison with the TPN + PF group		
	TPN + PF	TPN + LCT + PF	Low-LCT + PF	High-LCT + PF	TPN + LCT + PF	Low-LCT + PF	High-LCT + PF
Hexose	< 0.001	< 0.001	< 0.001	< 0.001	0.192	< 0.001	0.032
Hk1	< 0.001	< 0.001	< 0.001	< 0.001	0.501	0.052	0.987
Hexose-phosphate	< 0.001	< 0.001	< 0.001	< 0.001	0.424	0.798	0.252
Pfkm	< 0.001	< 0.001	< 0.001	0.013	0.403	0.29	0.038
Pfkfb3	0.988	0.232	0.109	0.568	0.096	0.135	0.351
Fbp1	< 0.001	< 0.001	0.001	< 0.001	0.708	0.087	0.613
Hexose-bisphosphate	< 0.001	< 0.001	0.007	< 0.001	0.563	0.027	0.584
GADP	< 0.001	< 0.001	< 0.001	< 0.001	0.323	0.506	0.462
DHAP	< 0.001	< 0.001	< 0.001	< 0.001	0.323	0.506	0.462
Phospho-glycerate	< 0.001	< 0.001	< 0.001	< 0.001	0.815	0.017	< 0.001
PEP	< 0.001	< 0.001	< 0.001	< 0.001	0.938	< 0.001	< 0.001
Pkm	< 0.001	< 0.001	< 0.001	< 0.001	0.501	0.597	0.963
Pck1	< 0.001	< 0.001	0.048	< 0.001	0.462	0.001	0.02
Pcx	< 0.001	< 0.001	< 0.001	< 0.001	0.963	0.772	< 0.001
Pyruvate	0.002	0.026	0.441	0.599	0.389	< 0.001	< 0.001

Pdk4	< 0.001	< 0.001	< 0.001	< 0.001	0.542	< 0.001	0.323
Acetyl-CoA	0.001	< 0.001	0.015	0.049	0.393	0.551	0.097
CoA	< 0.001	0.007	0.008	0.026	0.913	0.384	0.628
Lactate	< 0.001	< 0.001	< 0.001	< 0.001	0.938	< 0.001	< 0.001
Ldha	< 0.001	< 0.001	< 0.001	< 0.001	0.888	0.008	0.079
Alanine	< 0.001	< 0.001	0.026	0.233	0.913	< 0.001	< 0.001

Abbreviations: GADP: Glyceraldehyde 3-phosphate; DHAP: Dihydroxy-acetone phosphate; PEP: Phosphoenolpyruvate.

Supplementary Table S6: univariate statistical comparisons among groups for metabolites and mRNA levels shown in the heatmap of Fig. 8 in muscle tissue

	P-value of Wilcoxon signed-rank test in comparison with HC animals				P-value of Wilcoxon signed-rank test in comparison with the TPN + PF group		
	TPN + PF	TPN + LCT + PF	Low-LCT + PF	High-LCT + PF	TPN + LCT + PF	Low-LCT + PF	High-LCT + PF
Hexose	0.499	0.039	0.071	0.499	0.118	0.012	0.203
Hk1	< 0.001	< 0.001	0.003	< 0.001	0.888	0.095	0.767
Hexose-phosphate	0.061	0.189	< 0.001	0.002	0.791	< 0.001	< 0.001
Pfkm	0.245	0.845	0.037	0.21	0.134	0.088	0.279
Pfkfb3	0.002	< 0.001	0.008	0.94	0.888	< 0.001	0.027
Hexose-bisphosphate	0.94	0.988	< 0.001	< 0.001	> 0.9	< 0.001	< 0.001
GADP	0.053	0.049	< 0.001	0.011	0.938	< 0.001	< 0.001
DHAP	0.061	0.126	< 0.001	0.029	0.913	< 0.001	< 0.001
Phospho-glycerate	0.964	0.988	0.001	0.013	0.938	< 0.001	0.002
PEP	0.988	0.822	< 0.001	0.003	0.791	< 0.001	< 0.001
Pkm	0.558	0.358	0.806	0.822	0.815	0.281	0.355
Pyruvate	0.016	0.066	< 0.001	< 0.001	0.521	< 0.001	0.424
Pdha1	0.772	0.628	0.251	0.987	0.905	0.498	0.683
Pdk4	< 0.001	< 0.001	< 0.001	< 0.001	0.636	< 0.001	< 0.001
Acetyl-CoA	0.008	0.039	0.031	0.599	0.389	0.986	0.005

CoA	< 0.001	0.001	0.003	0.142	0.584	0.798	0.016
Lactate	< 0.001	0.002	< 0.001	< 0.001	0.888	< 0.001	0.044
Ldha	< 0.001	0.002	< 0.001	< 0.001	0.355	0.01	0.047
Cs	0.003	0.029	0.202	0.199	0.214	0.006	0.044
Citrate	< 0.001	0.029	< 0.001	0.046	0.389	0.036	0.203
Idh2	0.346	0.271	0.523	0.301	0.832	0.873	0.901
Cis-aconitate	0.061	0.578	< 0.001	0.111	0.308	0.002	0.988
α-ketoglutarate	< 0.001	< 0.001	< 0.001	0.845	0.308	0.187	< 0.001
Ogdh	< 0.001	< 0.001	0.302	0.007	0.743	0.005	0.203
NAD^+	0.006	< 0.001	0.567	0.049	0.406	0.011	0.265
NADH	0.098	0.098	0.182	0.663	0.988	0.721	0.017
ADP	0.374	0.034	0.481	0.284	0.134	0.081	0.719
ATP	0.271	0.21	< 0.001	0.01	0.767	< 0.001	0.002
Creatine	0.48	0.374	0.909	0.599	0.864	0.403	0.181
Phosphocreatine	0.845	0.578	0.029	0.036	0.501	0.088	0.068

Abbreviations: GADP: Glyceraldehyde 3-phosphate; DHAP: Dihydroxy-acetone phosphate; PEP: Phosphoenolpyruvate.

Supplementary Figures

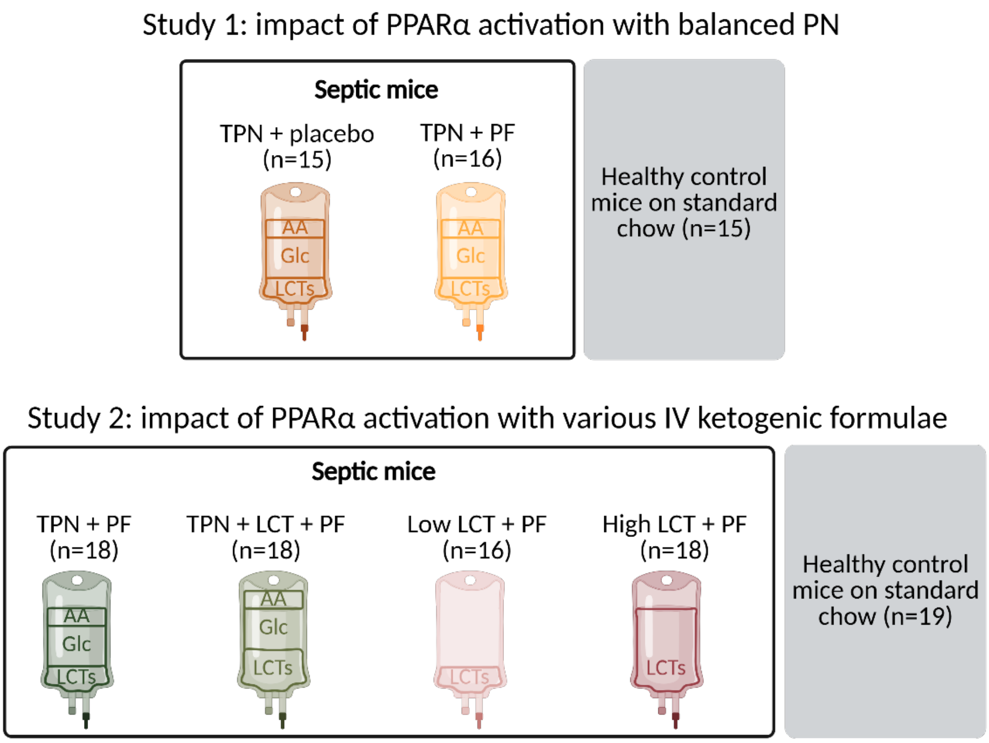

Supplementary Fig. S1 Schematic overview of study design and surviving animals in study 1 and 2. Abbreviations: TPN: total parenteral nutrition; PF: pemafibrate; IV: intravenous; LCT: long-chain triglyceride.

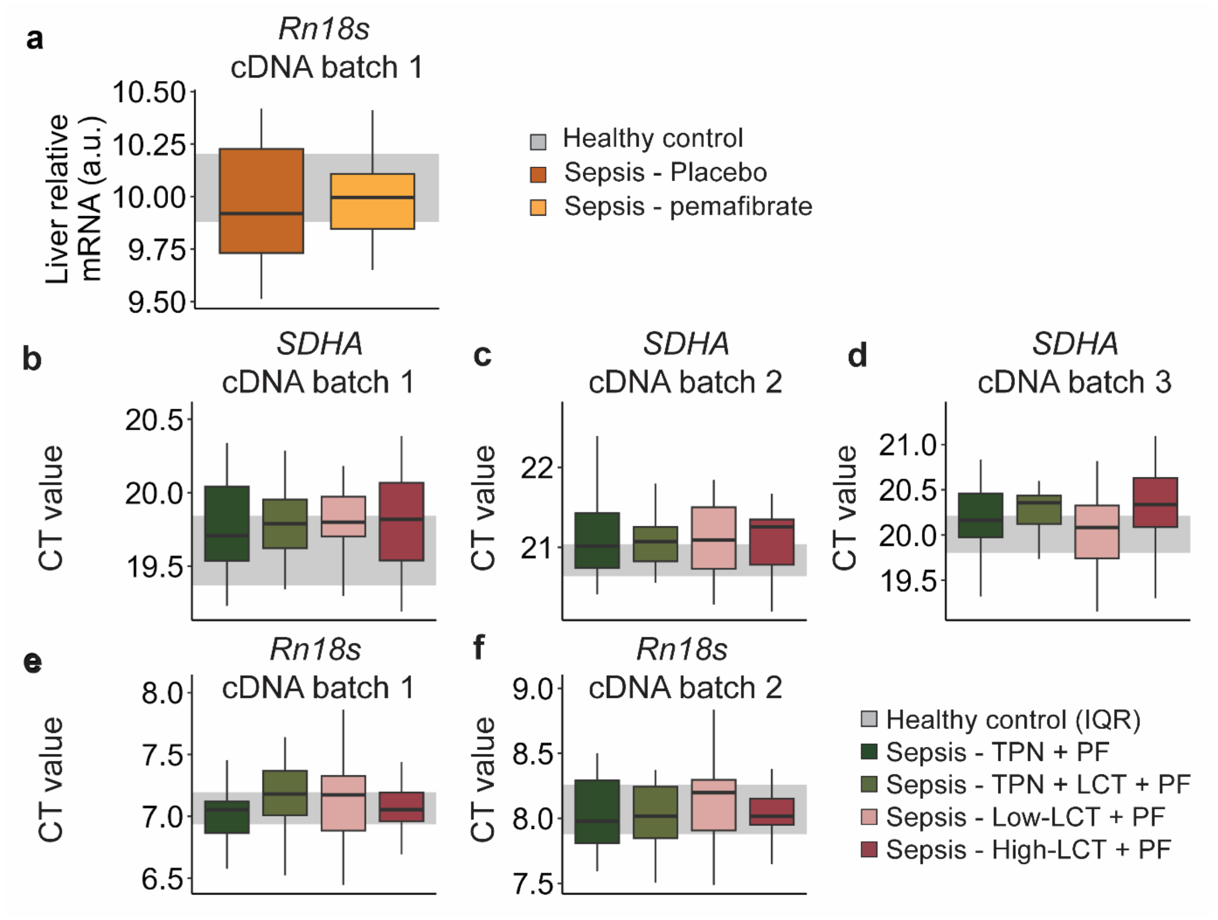

Supplementary Fig. S2

Housekeeping genes per cDNA batch for liver tissue (Rn18s, panel A) in study 1; and gastrocnemius (SDHA, panels B-D) and liver tissue (Rn18s, panels E-F) for study 2, Real-Time PCR analyses. Abbreviations: CT: cycle threshold; cDNA: copy deoxyribonucleic acid; TPN: total parenteral nutrition; PF: pemafibrate; IV: intravenous; LCT: long-chain triglyceride. Statistical significance is shown by */**/***/****: p<0.05/0.01/0.001/0.0001. Asterisks placed above boxplots denote comparisons with HCs, and the interquartile ranges of HC mice is shown in gray.

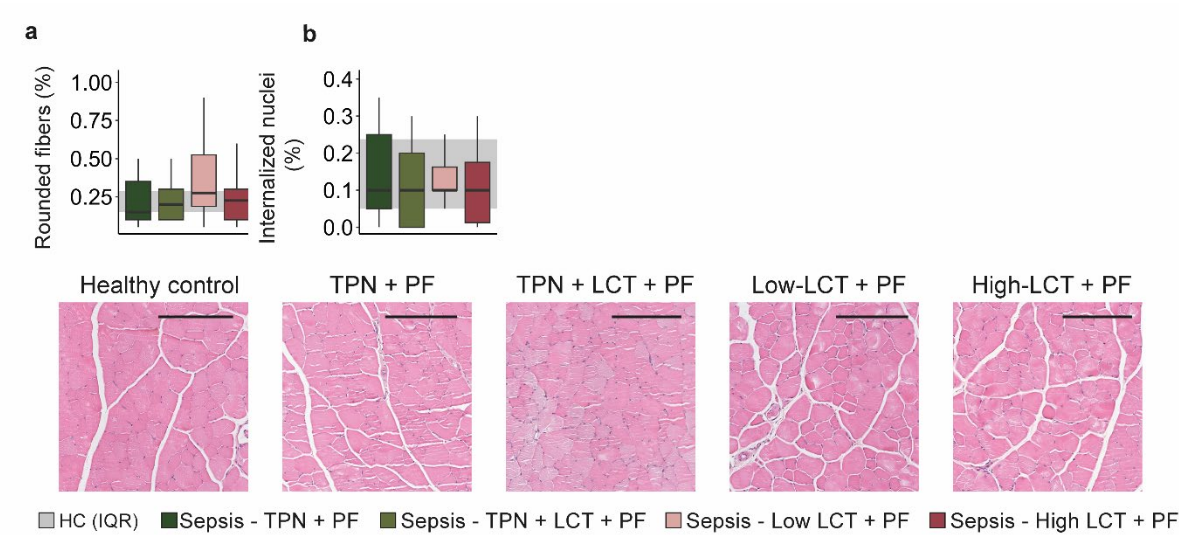

Supplementary Fig. S3

Muscle histological analysis by H&E staining, including (A) the number of rounded fibers and (B) internalized nuclei. Representative muscle histology images are shown per randomization group and black scale bars denote 200 µm. The interquartile range of HC mice is shown in gray.

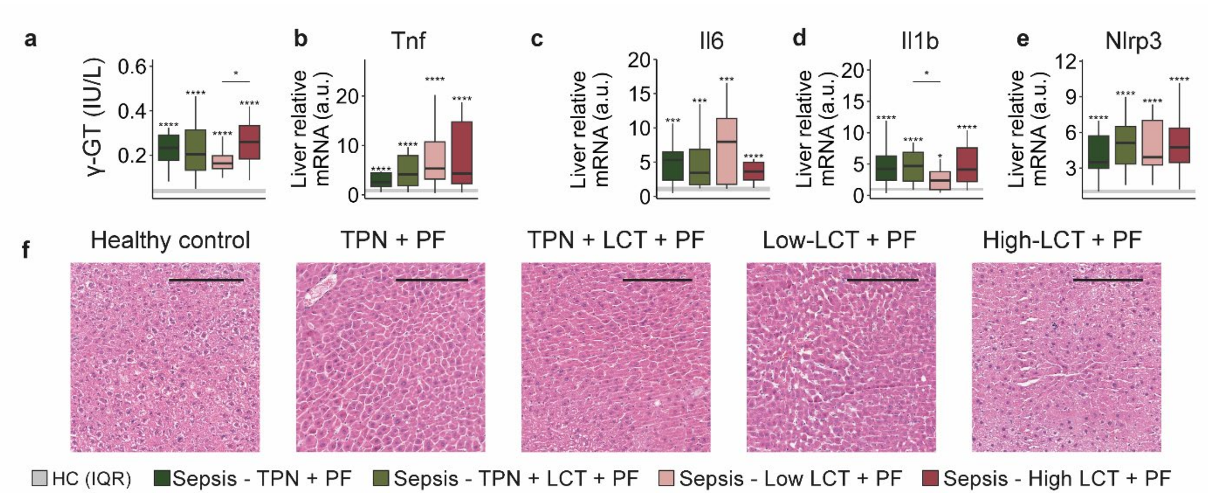

Supplementary Fig. S4

Markers of liver function, cytokine levels and histological integrity. (A) Plasma γ-GT levels after 5 days of sepsis. Relative mRNA expression of liver cytokines, including (B) TNF, (C) Il-6, (D) Il-1b and (E) Nlrp3. (F) Representative liver histology images are shown per randomization group and black scale bars denote 200 µm. Statistical significance is shown by */**/***/****: $p<0.05/0.01/0.001/0.0001$. Asterisks placed above boxplots denote comparisons with HCs, and the interquartile ranges of HC mice is shown in gray. Abbreviations: γ-GT: γ-glutamyltransferase

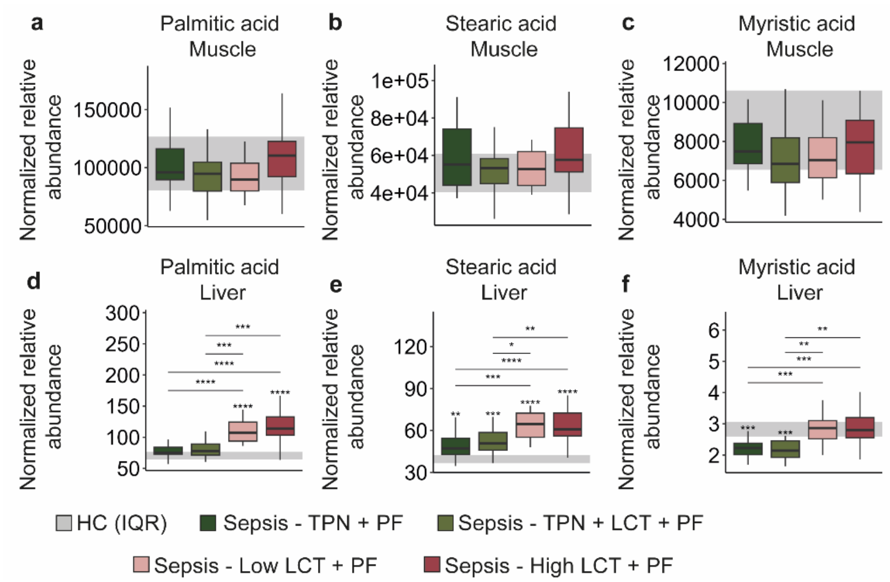

Supplementary Fig. S5

Free fatty acids including palmitic acid, stearic acid and myristic acid in muscle (panel A, B and C, resp.) and liver tissue (panel D, E and F, resp.). The interquartile range of HC mice is shown in gray. Statistical significance is shown by */**/***/****: p<0.05/0.01/0.001/0.0001. Asterisks placed above boxplots denote comparisons with HCs.

References

1. Goossens, C., et al., *Adipose tissue protects against sepsis-induced muscle weakness in mice: from lipolysis to ketones.* Crit Care, 2019. **23**(1): p. 236.
2. Pang, Z., et al., *MetaboAnalyst 6.0: towards a unified platform for metabolomics data processing, analysis and interpretation.* Nucleic Acids Research, 2024. **52**(W1): p. W398-W406.
